# Luteolin Disrupts Keratinocyte–Dendritic Cell Communication in Psoriasis by Targeting Rh Family C Glycoprotein

**DOI:** 10.1155/mi/9564209

**Published:** 2026-03-12

**Authors:** Qian Zhang, Yan-wei Gao, Cheng–cheng Feng, Liang Yang, Fang Chen, Shun Guo, Yuan-jie Liu, Qiu-ya Lu, Chen Ji, Hui Shen

**Affiliations:** ^1^ Department of Dermatology, Zhangjiagang TCM Hospital Affiliated to Nanjing University of Chinese Medicine, Zhangjiagang, 215600, Jiangsu, China, njucm.edu.cn; ^2^ People’s Hospital of Nanfeng Town, Zhangjiagang, 215600, Jiangsu, China; ^3^ Department of Dermatology, Affiliated Hospital of Nanjing University of Chinese Medicine, Jiangsu Province Hospital of Chinese Medicine, Nanjing, 210029, Jiangsu, China, jshtcm.com; ^4^ Department of Oncology, Affiliated Hospital of Nanjing University of Chinese Medicine, Jiangsu Province Hospital of Chinese Medicine, Nanjing, 210029, Jiangsu, China, jshtcm.com; ^5^ Nord Anglia School Suzhou, Xiangcheng, 215131, Jiangsu, China

**Keywords:** dendritic cells, keratinocytes, luteolin, psoriasis, RHCG, spatial domains

## Abstract

Psoriasis, a chronic inflammatory skin disease, arises from a dysregulated interaction between keratinocytes (KCs) and dendritic cells (DCs). We previously identified Rh family C glycoprotein (RHCG) as a key mediator of KC inflammation and DC activation. Here, we demonstrate that luteolin, a bioactive compound derived from the traditional Chinese formula cooling blood and detoxicating formula (CBDF), directly binds to RHCG, as confirmed by multiple computational methods and in vitro experiments. In vitro, luteolin suppressed RHCG expression in KCs, reducing CXCL14 secretion and subsequent DC activation. Spatial transcriptomics (STs) revealed that luteolin preferentially targets DC–enriched spatial domains and restores desmosomal protein expression (e.g., DSC2), which is dysregulated in psoriasis. In vivo, luteolin ameliorated psoriasis‐like inflammation in imiquimod‐induced mice, lowering Psoriasis Area and Severity Index (PASI) scores and normalizing pathological markers. Our findings indicate that luteolin disrupts KC–DC communication through multiple modes of action, thereby reversing tissue‐level pathology and demonstrating its potential as a targeted therapy for psoriasis.

## 1. Introduction

Psoriasis is an immune‐related and long‐term inflammatory disorder of the skin. The estimated global prevalence is 2%–3% [1]. China has more than 7 million patients, and about one‐third (34%) have moderate‐to‐severe disease [2]. Pathogenesis involves dysregulation of the IL‐23/Th17 axis [3], epidermal barrier defects and environmental triggers like obesity (body mass index [BMI] ≥30 increases progression risk by 2.3‐fold) [4]. Diagnosis relies on clinical evaluation, histopathology, and exclusion of differential diagnoses [5]. Treatment paradigms include topical agents (e.g., vitamin D3 analogs and corticosteroids) [6], phototherapy (narrowband‐ultraviolet B [NB‐UVB] as first‐line) [7], and systemic therapies (methotrexate [MTX], biologics like IL‐17/IL‐23 inhibitors) [8]. Despite advances, challenges persist: 30%–50% of patients experience inadequate responses or relapse due to comorbidities (e.g., psoriatic arthritis and cardiovascular disease) and psychosocial burdens (anxiety and depression). Biologic resistance and long‐term safety concerns (e.g., infection risks) further complicate management, necessitating personalized, and multidisciplinary approaches [9–12].

In psoriasis, keratinocytes (KCs) and dendritic cells (DCs) communicate closely. Their cross talk helps sustain ongoing inflammation and reinforces disease progression [13]. KCs, activated by environmental stressors (e.g., ultraviolet [UV] radiation radiation and microbial products) [14] or pro‐inflammatory cytokines (e.g., TNF‐α and IL‐17) [15], secrete chemokines (CCL20 and CXCL1‐3), and antimicrobial peptides (AMPs) like LL‐37, which recruit and activate plasmacytoid DCs (pDCs) and myeloid DCs (mDCs) [16]. Activated DCs, in turn, produce IL‐23 and IL‐12, polarizing naive T cells toward Th17 and Th1 phenotypes [15]. Th17‐derived IL‐17A/F and TNF‐α further stimulate KCs, amplifying NF‐κB and STAT3 signaling to sustain epidermal hyperplasia and neutrophil infiltration [17]. This cross talk establishes a feedforward mechanism, as evidenced by single‐cell RNA sequencing (scRNA‐seq) showing upregulated DC–KC signaling pathways in lesional skin. Targeting this axis (e.g., anti‐IL‐23 biologics) disrupts inflammation, underscoring its therapeutic relevance [18]. However, contextual dependencies—such as tissue‐specific DC subsets and microenvironmental cues—remain incompletely understood, necessitating further mechanistic studies.

Traditional Chinese medicine (TCM) has gained increasing recognition in psoriasis management due to its multitarget effects and holistic approach, particularly for mild‐to‐moderate cases [19]. Herbal formulations like Indigo naturalis (Qing Dai) [20] and Radix Paeoniae Rubra (Chi Shao) exhibit anti‐inflammatory properties via inhibition of NF‐κB and STAT3 pathways, while acupuncture modulates immune dysregulation through neuroimmune cross talk [21, 22]. Recent advances in component analysis—such as UPLC‐MS/MS and network pharmacology—have enabled the identification of active constituents, including indirubin (from *Indigofera tinctoria*) and paeoniflorin. Functionally, these compounds have been linked to decreased Th17/IL‐23 axis activity [23]. Concurrently, TCM modernization efforts—standardization of herbal extracts, quality control via DNA barcoding, and integration with systems biology—are driving evidence‐based validation [24]. However, challenges persist in elucidating synergistic mechanisms of multiherb formulations and addressing variability in herbal sourcing. Emerging studies leveraging single‐cell omics and AI–driven target prediction are poised to bridge this gap, reinforcing TCM’s role in personalized and integrative psoriasis therapies.

Our recent work has identified Rh family C glycoprotein (RHCG) as a disease‐relevant regulator in psoriasis and has mapped its role across multiple omics layers. In our earlier study [25], we identified RHCG as a hub gene associated with KC differentiation programs and DC maturation. In our subsequent studies [26, 27], we further showed that RHCG is hypoxia‐responsive and can enhance KC inflammatory activation, including increased CXCL14 output, which promotes DC maturation through the CXCL14–CXCR4 axis.

Mechanistically, RHCG drives KC inflammation by upregulating S100A proteins and CXCL14, which activates DCs via CXCL14–CXCR4 signaling, enhancing DC maturation (LAMP3^+^). This connection suggests that hypoxic stress may tune inflammatory responses by regulating RHCG–mediated chemokine secretion from KC, which in turn could promote DC activation. Importantly, an independent study also reported that RHCG overexpression drives KC proliferation and pro‐inflammatory cytokine release in psoriasis models, providing external support for RHCG’s pathogenic role [28]. Together, these studies establish RHCG as a key node linking epidermal stress responses to KC–DC communication in psoriasis. However, it remains unclear whether this RHCG–centered pathway can be modulated by a defined small molecule in a way that disrupts downstream KC–DC signaling.

Building on these findings, we are now prioritizing the elucidation of cooling blood and detoxicating formula’s (CBDF’s) bioactive components and molecular mechanisms.

In the present study, we systematically investigated the bioactive components of CBDF and their therapeutic mechanisms in psoriasis. Through in silico analysis, including molecular docking and binding energy calculations, we identified luteolin as the most promising candidate due to its lowest binding energy with RHCG. Subsequent molecular dynamics simulations and thermoshift assays provided definitive evidence of direct luteolin–RHCG interaction. In vitro experiments demonstrated that luteolin suppresses KC inflammation by downregulating RHCG expression, thereby attenuating DC activation via reduced CXCL14–CXCR4 signaling. Integrating spatial transcriptomics (STs) with in vivo models, we revealed that luteolin reprograms the spatial architecture of KC–DC coordination domains, while restoring desmosomal protein expression (e.g., desmoglein‐1/3) diminished in psoriatic lesions. Overall, this work extends our prior RHCG framework by showing that luteolin can modulate the RHCG–centered KC–DC inflammatory axis, providing a preclinical rationale for mechanism‐guided luteolin‐based interventions.

## 2. Materials and Methods

### 2.1. Molecular Docking

Molecular docking was used to evaluate the possible binding of RHCG to the core targets. The RHCG structure files (PDB format) were downloaded from the RCSB Protein Data Bank (RCSB PDB) [29]. Docking results were then analyzed and visualized using PrankWeb [30]. Ligand SMILES files of five active ingredients in CBDF were downloaded from PubChem.

### 2.2. Protein–Protein Interaction (PPI) Network Construction and Enrichment Analysis

A PPI network for luteolin‐related targets was generated using STRING [31]. The interaction information were extracted and imported into Cytoscape for network construction and analysis. Functional enrichment was then carried out with the R package clusterProfiler [32].

### 2.3. Molecular Dynamics Simulation Analysis

Protein–ligand complex flexibility was evaluated using CABS‐flex v2.0 [33] and the iMOD server (iMODS) [34]. For RHCG, CABS‐flex was applied to estimate structural fluctuations, reported as root‐mean‐square fluctuation (RMSF). The run length was set to 10 ns, and the remaining options were kept at default. RMSF curves were generated from the resulting trajectory (or from an NMR ensemble) using the default procedure. We then submitted the docked RHCG–luteolin PDB structures to iMODS to evaluate complex stability and collective motions. iMODS–based outputs included deformability, B‐factor, eigenvalue, variance, covariance map, and elastic network features, all calculated under default settings.

### 2.4. scRNA‐seq Analysis

We downloaded scRNA‐seq data from the National Center for Biotechnology Information (NCBI) Gene Expression Omnibus (GEO) database under accession GSE151177 [35]. The dataset contains 13 lesional skin samples from patients with psoriasis and five normal skin samples from healthy volunteers. Sequencing data preprocessing followed established methodologies from a prior study. Cell exclusion criteria comprised: (1) expression of fewer than 200 genes, and (2) a mitochondrial UMI content exceeding 40%. Putative doublets were subsequently removed using DoubletFinder with default parameters [36]. After preprocessing, we integrated the qualified datasets with the Harmony algorithm [37] to support subsequent dimensionality reduction and clustering. Dimensionality reduction was performed by principal component analysis (PCA) using default parameters, and the top 30 principal components were selected for clustering. Clusters were identified with the FindCluster function (resolution = 0.3) and visualized using uniform manifold approximation and projection (UMAP). Cluster markers were identified with FindAllMarkers using the following thresholds: adjusted *p*  < 0.05, log_2_ fold change (log_2_ FC) > 0.25, and min.pct >0.25. Cell types were annotated based on canonical marker genes reported in the literature. For subclustering of the cell type of interest, we identified subcluster‐enriched marker genes using the FindAllMarkers function [38] with the following criteria: adjusted *p*  < 0.05, log_2_ FC >0.1, and min.pct >0.1. Figures were generated using the scplotter package (v0.5.3) [39].

### 2.5. Computational Framework for the Targeted Spatial Domain of Luteolin

STs data were obtained from the CROST database, including four psoriasis samples: VISDS001018, VISDS001019, VISDS001020, and VISDS001021 [40].

Seurat (v5.0) was used for initial preprocessing of the STs dataset [38]. Raw count matrices were processed with SCTransform to generate variance‐stabilized values, followed by dimensionality reduction with RunPCA. Deconvolution outputs available from the CROST database were then retrieved, and spatial domain segmentation was performed using BayesSpace (v1.6.0) [41]. Within BayesSpace, we applied spatialPreprocess for log‐normalization and PCA [38] and used qTune to determine the optimal clustering setting. Spatial domains were inferred with spatialCluster (*q* = 8, *γ* = 3, nrep = 10,000) based on a Markov random field model that incorporates spatial coordinates. Finally, results were integrated in SpaTopic (v0.1.0) [42]. Using CellTopic, BayesSpace domain labels were merged with CROST‐derived cell‐type proportions, and domain‐level enrichment was summarized by ranking cell types according to their mean proportions to identify dominant populations.

Finally, luteolin target‐gene signatures were scored in the ST dataset using six gene set scoring/enrichment approaches. To evaluate domain specificity, we compared mean signature scores across spatial domains and defined “targeted” domains as those with scores above the 90th percentile relative to background. Plots were generated using the semla package (v1.3.0).

Domain–domain communication was inferred using ligand–receptor annotations from CellChat [43], and the results were analyzed and visualized accordingly.

### 2.6. Bulk‐Seq Analysis

Three bulk RNA‐seq datasets of psoriasis, along with their corresponding clinical data, were retrieved from the GEO database. Specifically, dataset GSE13355 [44] includes 64 normal skin samples from healthy donors (NN), 58 nonlesional skin samples from psoriasis patients (PN), and 58 lesional skin samples from psoriasis patients (PP). Dataset GSE14905 [45] comprises 21 NN, 28 PN, and 33 PP samples. Dataset GSE30999 [46] contains 85 PN and 85 PP samples, respectively. All datasets were generated using the Affymetrix Human Genome U133 Plus 2.0 Array platform (GPL570) [47]. For subsequent analysis, data normalization was performed using the “normalizeBetweenArrays” function from the “limma” R package (v3.60.3) [48].

### 2.7. Experimental Materials

All chemical compounds and antibodies used in this study are listed in Supporting Information 1: Table S1. Antibody dilutions/concentrations were selected according to the manufacturers’ instructions or prior publications. Information for all supporting figures is also provided in supporting information section.

### 2.8. Cell Culture Conditions

HaCaT, a spontaneously immortalized human KC line, was purchased from Wuhan Pu‐nuo‐sai Life Technology Co., Ltd. (Wuhan, China). Cell line information was as follows: HaCaT (official name: HaCaT; species: *Homo sapiens*; sex: male; tissue of origin: skin from a 62‐year‐old male; RRID: CVCL_0038). HaCaT cells were maintained in minimum essential medium (MEM) supplemented with 10% (*v*/*v*) fetal bovine serum (FBS) and 1% penicillin/streptomycin. Immature human peripheral blood DCs (Catalog Number CP‐H179A) were obtained from the same supplier. These primary DCs (species: *Homo sapiens*; tissue of origin: peripheral blood) were cultured in basal medium containing 10% (*v*/*v*) FBS, 1% penicillin/streptomycin, and DC growth factors (complete medium for human peripheral blood DCs). All cell cultures were maintained under standard conditions (37°C, 5% CO_2_, humidified atmosphere).

### 2.9. Cell Line Authentication and Quality Control

The HaCaT cell line was authenticated using short tandem repeat (STR) profiling, confirming the identity and integrity of the cells. This cell line has not been previously reported as misidentified or contaminated. Mycoplasma contamination was assessed using PCR–based detection assays, and no contamination was detected in the cultured cells. The human peripheral blood DCs are primary cells and, therefore, not subject to authentication requirements beyond the supplier’s quality control.

### 2.10. Luteolin Treatment and Cell Viability Assay

Luteolin was prepared as a dimethyl sulfoxide (DMSO) stock solution. During the exponential growth phase, HaCaT cells were exposed to luteolin at 0, 1, 10, 25, 50, or 100 μM. The vehicle control received an equal volume of DMSO, with the final DMSO concentration kept at ≤0.1%. Treatments were carried out for 24, 48, or 72 h, and each time point was analyzed independently. Cell viability was measured using the cell counting kit‐8 (CCK‐8) assay. At each time point, 10 μL of CCK‐8 reagent was added per well and incubated at 37°C for 1–2 h. Absorbance was read at 450 nm using a microplate reader (EL800, Bio‐Tek, USA), and OD values refer to the signal at 450 nm. Cell viability was calculated relative to the corresponding vehicle control.

### 2.11. Cellular Thermal Shift Assay (CETSA)

CETSA assays were carried out according to a published protocol [49]. Guided by the CCK‐8 assay, we selected a luteolin treatment that showed no obvious effect on HaCaT proliferation for CETSA (10 μM, 24 h). Cells were harvested, washed, and resuspended in phosphate‐buffered saline (PBS). Samples were portioned into aliquots and subjected to a temperature gradient for 3 min in a PCR plate. A protease inhibitor cocktail was then added, and cell disruption was achieved through three liquid nitrogen–based freeze–thaw cycles with a heat block. After lysis, samples were centrifuged at 20,000 × *g* for 20 min at 4°C to remove aggregated/precipitated proteins. The resulting supernatants (soluble fractions) were collected for analysis. Soluble proteins were resolved by SDS‐PAGE and analyzed by western blotting (WB). WB was performed as previously described [50]. Band intensities were measured in ImageJ. For thermal stability analysis, signal at each temperature was normalized to the value at the lowest temperature, and the normalized values were used to plot stability curves. Experiments were repeated with at least three biological replicates.

### 2.12. Hematoxylin/Eosin (HE) Staining

Skin tissues were collected from 30 psoriasis cases and 30 normal controls. Specimens were fixed in 10% formaldehyde for 24 h, dehydrated in graded ethanol (70%–100%), paraffin‐embedded, and cut into 4 µm sections. Tissue sections were deparaffinized in xylene and rehydrated through a graded ethanol series (100%–50%). Following standard hematoxylin (5 min) and eosin (1 min) staining, slides were mounted and coverslipped. Images were acquired using a Nikon Eclipse Ni‐E upright microscope.

### 2.13. Plasmid‐Based Overexpression of RHCG in HaCaT Cells

Using Lipofectamine 3000, HaCaT KCs were transfected with an endotoxin‐free plasmid encoding human RHCG, following the manufacturer’s protocol. Cells were maintained in MEM + 10% FBS at 37°C, 5% CO_2_ and seeded to approximately 60%–70% confluence 24 h before transfection. For 6‐well plates, 1.5–2.0 µg DNA was diluted in Opti‐MEM with P3000 enhancer, mixed with Lipofectamine 3000, incubated 15 min at room temperature (RT, 20–25°C), and added dropwise; medium was replaced after 8 h. Where stable expression was required, puromycin (2 µg/mL, determined by a prior kill curve) was applied 48 h posttransfection for 3 days and then maintained at 1.0 µg/mL. Negative control (NC) plasmid was included. RHCG overexpression was confirmed by WB (Supporting Information 1: Figure S1).

### 2.14. Multiplex Immunofluorescence (mIF)

For multicolor tissue immunofluorescence, we used a tyramide signal amplification (TSA) workflow to increase detection sensitivity. Paraffin sections were deparaffinized and rehydrated through graded ethanol. Endogenous peroxidase was quenched with 3% H_2_O_2_ for 30 min. Following heat‐induced epitope retrieval (HIER), sections were blocked with 3% bovine serum albumin (BSA) for 1 h. Sections were incubated with primary antibodies at 4°C overnight. After washing, HRP–conjugated anti‐rabbit secondary antibodies were applied for 1 h at RT, and fluorescent tyramide was used for signal development. To enable additional markers while limiting background, a second cycle of HIER, BSA blocking, and antibody staining was carried out. Nuclei were counterstained with DAPI. Whole‐slide fluorescent images were acquired using the Pannoramic SCAN system (3DHISTECH, Hungary) at high resolution, and the same acquisition parameters were used for all specimens.

### 2.15. WB

Following extraction in RIPA lysis buffer containing protease inhibitors, protein concentrations were determined using the Bradford assay. Samples (20 µg protein/lane) were resolved by 10% SDS‐PAGE and transferred to PVDF membranes via semi‐dry blotting. Prior to overnight incubation with primary antibodies at 4°C, membranes were blocked for 1 h at RT in 5% BSA/TBST. After three TBST washes, membranes were incubated with HRP–conjugated secondary antibodies for 1 h at RT. Chemiluminescence was developed with ECL and imaged on an ImageQuant LAS 500 instrument (Cytiva, USA). Densitometry was performed in ImageJ, normalized to β‐actin or GAPDH from the same membrane/exposure, and expressed relative to the control mean. At least three independent biological replicates were analyzed.

### 2.16. Establishment of Coculture Units

KC–DC cross talk was examined in a noncontact transwell system (0.4 μm pore size). HaCaT cells were transfected with either a negative‐control plasmid (NC) or an RHCG overexpression construct (oe‐RHCG). To model psoriasis‐like inflammatory stimulation, HaCaT cells were exposed to the M5 cytokine cocktail [51] (IL‐6, IL‐17, TNF‐α, IFN‐γ, and oncostatin M; each at 10 ng/mL) for 24 h. The M5 medium was then removed, and HaCaT cells were treated for another 24 h with luteolin (10 μM) or MTX (50 ng/mL) as a positive control. To avoid direct compound exposure to DCs, luteolin/MTX was added only during the HaCaT pretreatment step. Before coculture, HaCaT cells were washed and replaced with fresh drug‐free medium. For coculture, HaCaT cells (1 × 10^5^ cells/well) were seeded in the upper inserts and DCs (5 × 10^4^ cells/well) in the lower wells of a 6‐well transwell plate, and cells were maintained for 24 h to allow paracrine signaling across the membrane [52]. After coculture, DCs were collected for WB, and culture supernatants were saved for ELISA.

### 2.17. ELISA Assay

Cytokine levels in the coculture supernatants (CXCL14, IL‐23, and IL‐6) were measured using commercial ELISA kits. Sample media were clarified by centrifugation at 3500 rpm for 10 min at RT to remove cell debris, followed by collection of the supernatant for testing. ELISAs were carried out according to the manufacturers’ protocols. In brief, microplates precoated (or coated) with cytokine‐specific capture antibodies were incubated as instructed, followed by washing and blocking to reduce nonspecific binding. After adding samples and standards, plates were incubated to capture cytokines on the immobilized antibodies. Detection was carried out using biotin‐labeled antibodies followed by streptavidin–HRP. Signal was developed with substrate, and absorbance was measured at 450 nm on a microplate reader (Bio‐Tek ELX800, Winooski, VT). Cytokine levels were determined by interpolation from kit‐generated standard curves.

### 2.18. Psoriasis‐Like Animal Model Establishment With Broussonetia papyrifera and Luteolin Administration Protocol

Twenty‐four specific pathogen‐free (SPF) female C57BL/6J (B6) mice (8 weeks; 18–22 g) were included. Mice were purchased from Henan Scripps Biotechnology Co., Ltd. (License Number SCXK [Yu] 2020‐0005) and maintained at 22 ± 2°C with 55% ± 10% humidity under a 12 h light/12 h dark cycle, with standard chow and water available ad libitum. All procedures were approved by the Animal Ethics Committee of Zhangjiagang Hospital of Traditional Chinese Medicine (Approval Number KY‐2023‐11‐09‐01); all animal experiments followed the NIH Guide for the Care and Use of Laboratory Animals. We assigned animals to four groups randomly (*n* = 6): IMQ model, blank control, luteolin, and MTX. A 2 cm × 3 cm dorsal area was shaved prior to modeling. To induce psoriasis‐like inflammation, mice in the model and treatment groups were treated daily for 7 days via topical application of 5% imiquimod cream (62.5 mg/day). During the same period, MTX was administered by gavage (0.2 mL/day; 1 mg/kg), and luteolin was delivered intraperitoneally (0.2 mL/day; 50 mg/kg). For the luteolin formulation, 50 mg luteolin was sonicated in 0.5 mL DMSO; 2 g SBE‐β‐CD was dissolved in saline and adjusted to 10 mL; then 0.5 mL of the luteolin–DMSO stock was combined with 9 mL of the SBE‐β‐CD solution and brought to 10 mL, giving a final luteolin concentration of 5 mg/mL with 5% DMSO. Blank controls received topical Vaseline (matched amount) and distilled water by gavage. Lesion severity was evaluated daily using Psoriasis Area and Severity Index (PASI) scoring (erythema, scaling, and infiltration). At day 7, mice were anesthetized with sodium pentobarbital and sacrificed by cervical dislocation, and skin and serum were collected for subsequent analyses.

### 2.19. Statistical Analysis

Data were analyzed using GraphPad Prism software (version 10.0) and are expressed as mean ± standard deviation (SD) unless noted otherwise. Specific sample sizes (*n*) and statistical methods are provided in the respective figure legends. Intergroup comparisons involving more than two groups were conducted using one‐way ANOVA, with Tukey’s post hoc test applied for multiple comparisons as appropriate. For omics‐related analyses, *p* values were corrected for multiple testing as detailed in the relevant Section 2, and adjusted *p* values are provided when appropriate. When available, exact *p* values are annotated on the figures.

## 3. Results

### 3.1. Molecular Docking Identifies Luteolin as the Key Component in CBDF for Targeting RHCG

Molecular docking was performed to estimate the binding affinities between RHCG and five major constituents of CBDF. The RHCG structure (PDB ID: 3HD6) was obtained from the RCSB PDB. As shown in Figure 1A,B, the predicted binding energies for all five compounds were below −6.0 kcal/mol. Among these candidates, luteolin yielded the most favorable docking score, suggesting a relatively stronger interaction with RHCG in this in silico analysis.

Figure 1Identification of luteolin as the most promising active ingredient in cooling blood and detoxicating formula (CBDF) and related functional enrichment. (A) Molecular docking of molecule structures and binding sites between RHCG and six active ingredients. (B) Binding energy heat map. Orange indicates strong binding activity, and blue indicates weak binding activity. (C) Protein–protein interaction (PPI) network illustrates the interactions among potential target genes of luteolin involved in the treatment of psoriasis. Nodes correspond to genes, with their sizes proportional to their degree centrality—larger nodes indicate higher connectivity. Orange nodes denote the predicted target genes of luteolin, whereas blue nodes represent genes associated with these predicted targets. See also Supporting Information 1: Table S1 and Supporting Information 2: S2–S8. (D) Gene Ontology (GO) and Kyoto Encyclopedia of Genes and Genomes (KEGG) pathway enrichment analysis of the potential target genes of luteolin.

**Figure:**
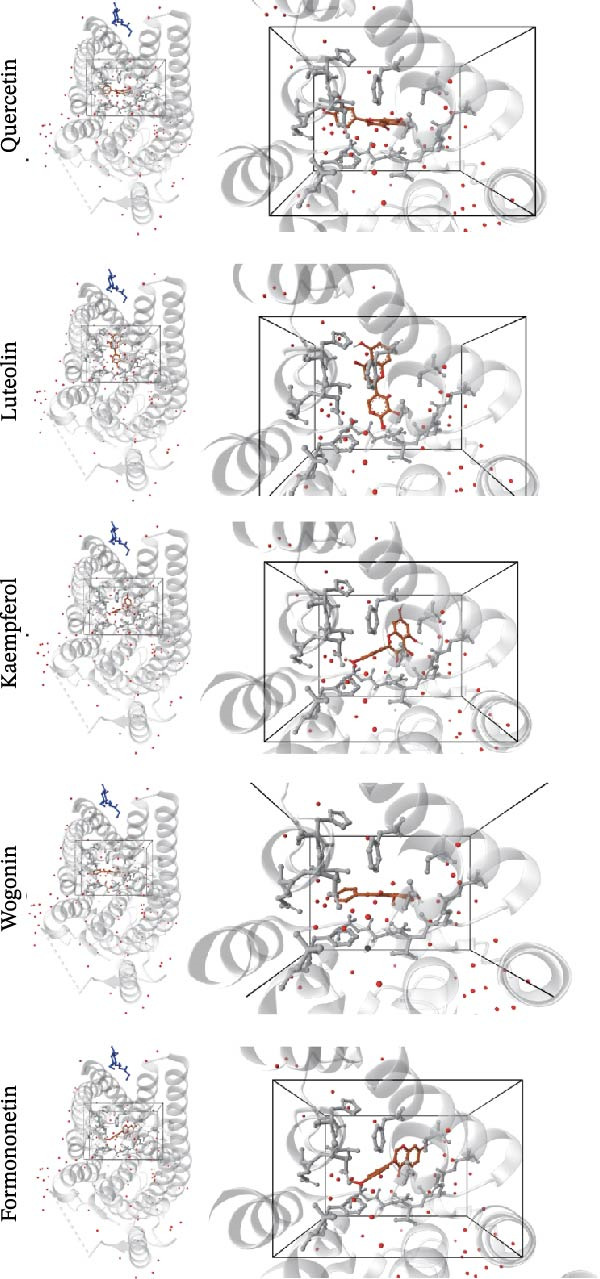
(A)

**Figure:**
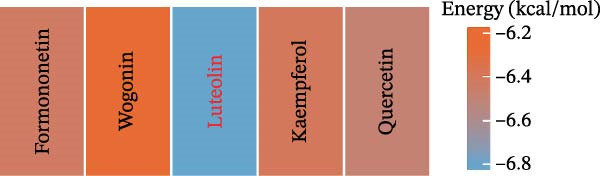
(B)

**Figure:**
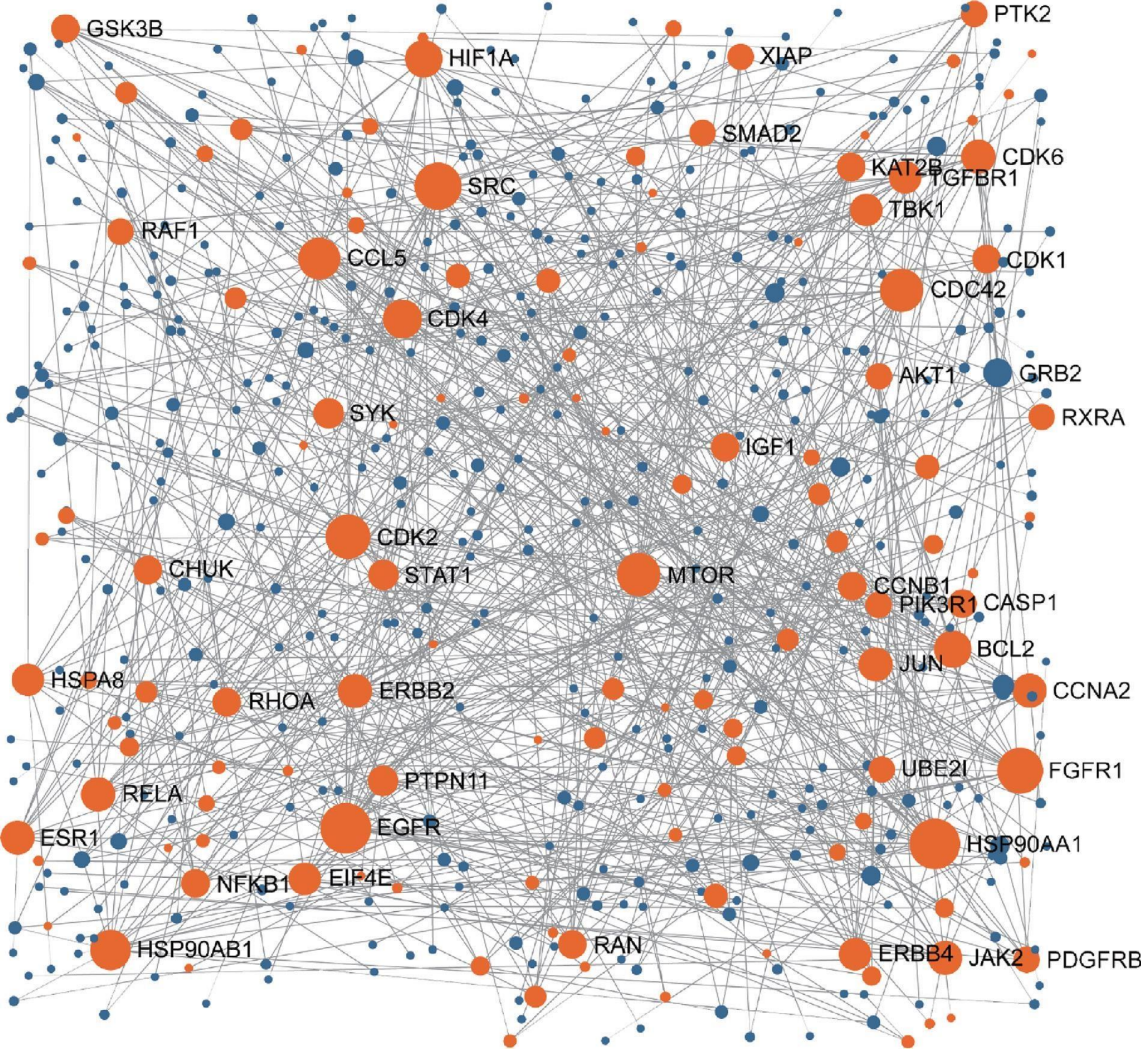
(C)

**Figure:**
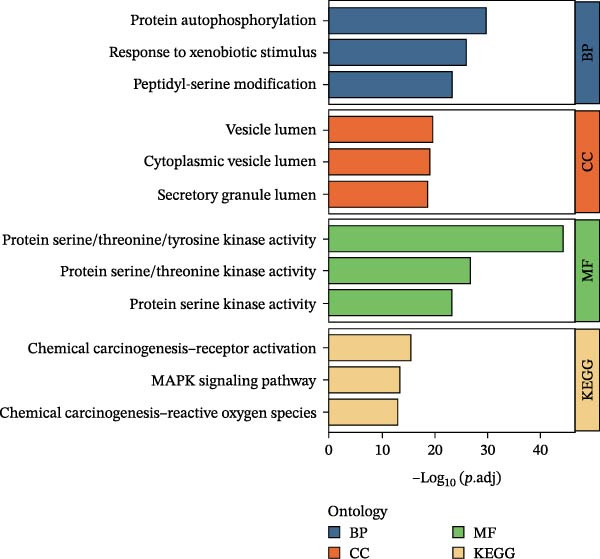
(D)

Next, we focused our research on luteolin. After exploring the D3CARP [53], PharmMapper [54], SEA [55], STITCH [56], SuperPred [57], and SwissTargetPrediction [58], 564 targets without duplicate values were obtained (Supporting Information 2: Tables S2–S9). To explore the potential therapeutic mechanisms of luteolin, we load these candidate targets into the STRING database to generate a target network. Target nodes were screened according to the principle of network topology. These targets play a key role in the whole network and may be the key downstream factors of RHCG for treating psoriasis. The important targets were SRC, HSP90AA1, EGFR, FGFR1, MTOR, and so on (Figure 1C). GO enrichment analysis (Figure 1D) showed that the candidate luteolin targets were enriched in several Gene Ontology terms. For biological processes, the main terms included protein autophosphorylation, response to xenobiotic stimulus, and peptidyl‐serine modification. For cellular components, the top terms were vesicle lumen, cytoplasmic vesicle lumen, and secretory granule lumen. For molecular functions, kinase‐related terms were dominant, including protein serine/threonine/tyrosine kinase activity and protein serine/threonine kinase activity. KEGG enrichment analysis (Figure 1D) linked these targets to multiple pathways, with leading pathways including chemical carcinogenesis‐receptor activation, the MAPK signaling pathway, and chemical carcinogenesis–reactive oxygen species.

### 3.2. MD Simulation Combined With CETSA Confirmed the Binding of Luteolin and RHCG

Next, we examined the putative interaction between luteolin and RHCG using molecular dynamics–based flexibility analysis. This approach helps assess complex stability and potential ligand‐associated structural changes. Residue‐level mobility was quantified by RMSF calculated with CABS‐flex, where higher RMSF values reflect greater flexibility and lower values indicate more constrained motion. Using the docked PDB structure as input under default settings, CABS‐flex generated 10 representative models and the corresponding RMSF plot (Figure 2A,B and Supporting Information 2: Table S10). In chain A of RHCG, the largest fluctuation was observed at residue 34 (RMSF = 4.483 Å), whereas residue 256 showed the lowest fluctuation (RMSF = 0.053 Å).

Figure 2RHCG is a target of luteolin. (A, B) Multimodel superimposed simulated structure and molecular dynamics (MD) simulation showing the root‐mean‐square fluctuation (RMSF) profiles of RHCG. See also Supporting Information 2: Table S9. (C–I) Upshots of molecular dynamics simulation in iMODS for RHCG–luteolin complex. Deformability (C), B‐factor (D), variance (E), eigenvalues (F), covariance plot (G), elastic network model (H), and residue–residue interaction (I). (J) CCK‐8 assay was used to measure cell viability of HaCaT cells treated with luteolin at concentrations of 0, 1, 10, 25, 50, and 100 μM. Data represent absorbance (OD value at 450 nm) measured at 24, 48, and 72 h. (K) Western blot reveals dose‐dependent suppression of RHCG by luteolin (0, 2, 4, and 8 μM) in HaCaT cells. GAPDH is the loading control. (L) Cellular thermal shift assay (CETSA)–WB of HaCaT cell lysates showing RHCG thermal stabilization in the presence of luteolin (10 μM) compared with dissolved in dimethyl sulfoxide (DMSO). RHCG band intensities were quantified, normalized to 37°C (set to 1), and fitted with a Boltzmann sigmoidal model to estimate melting temperatures (*T*_m_). Luteolin increased the apparent *T*_m_ of RHCG (*ΔT*_m_ = +7.4 ± 0.5°C; *n* = 3; paired *t*‐test *p* = 0.0039). Statistical analysis: All experiments were independently repeated three times. Data are presented as mean ± SD, and statistical significance was assessed using one‐way ANOVA with Tukey’s post hoc test. Exact *p* values are labeled on the figure.

**Figure:**
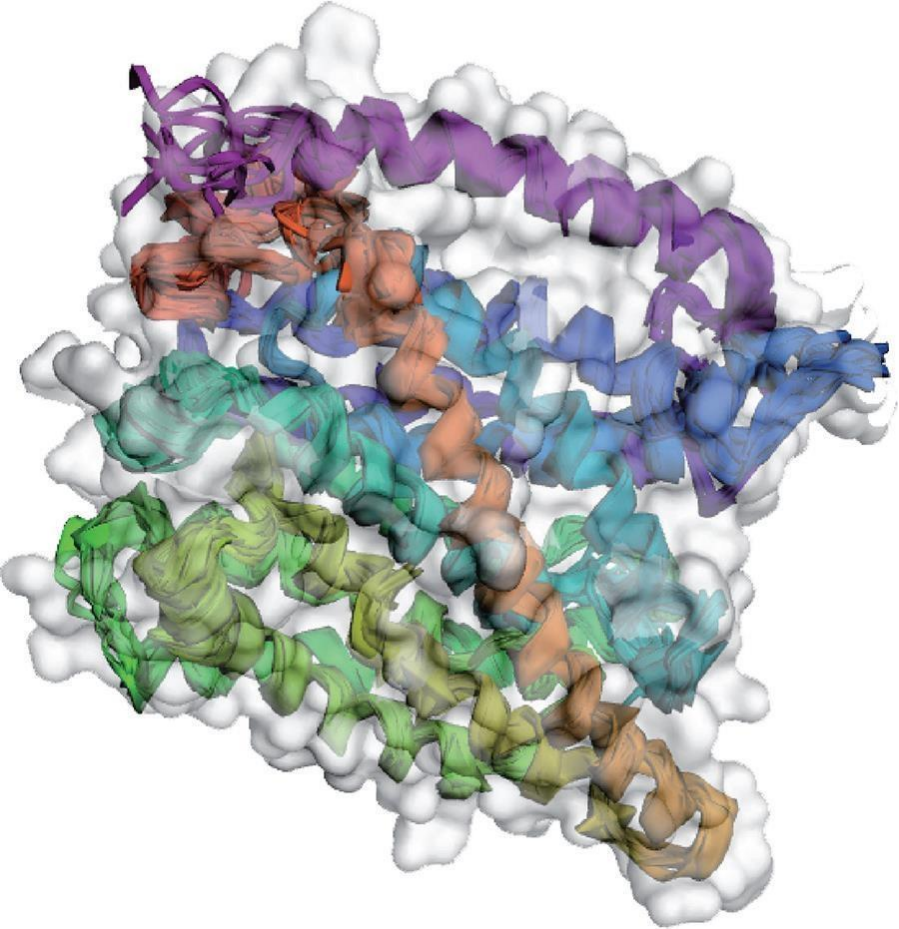
(A)

**Figure:**
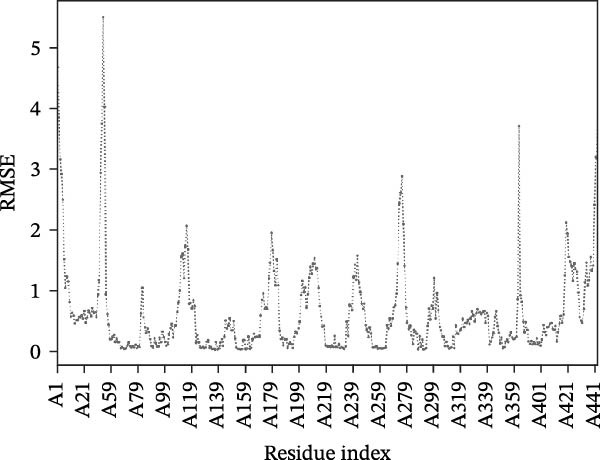
(B)

**Figure:**
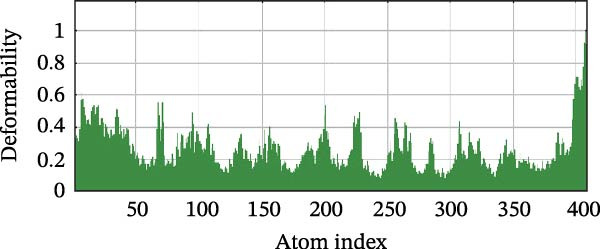
(C)

**Figure:**
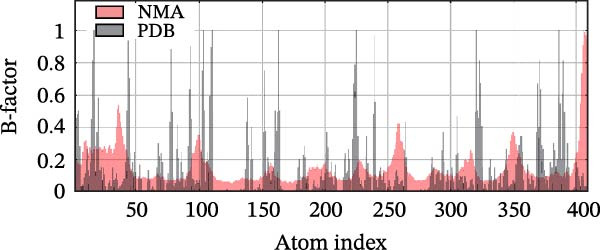
(D)

**Figure:**
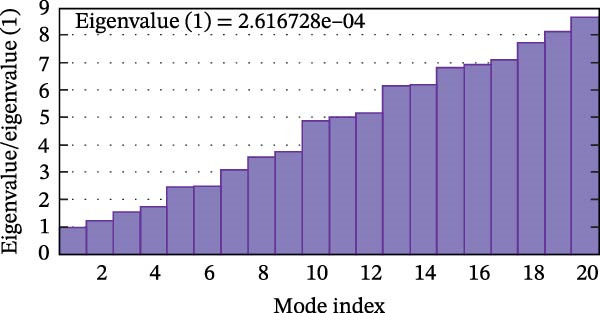
(E)

**Figure:**
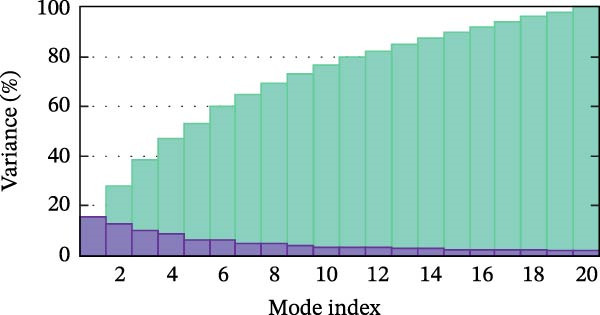
(F)

**Figure:**
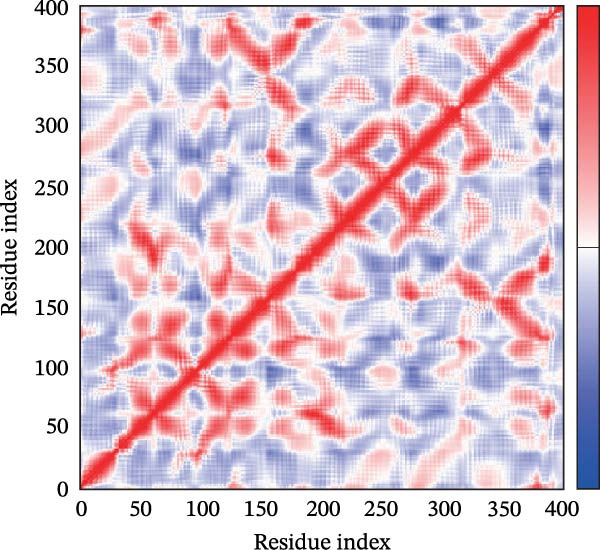
(G)

**Figure:**
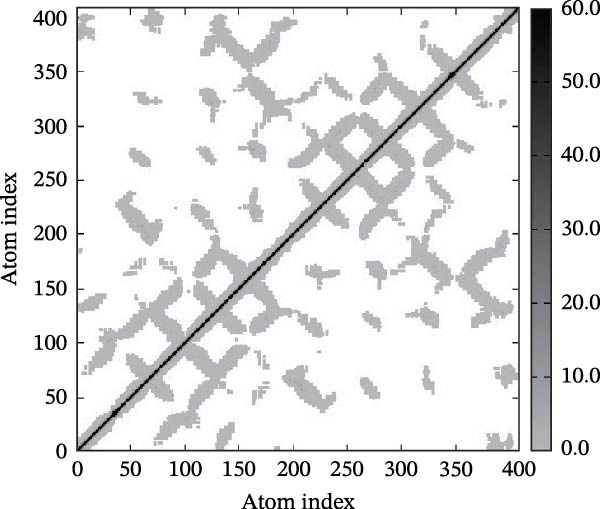
(H)

**Figure:**
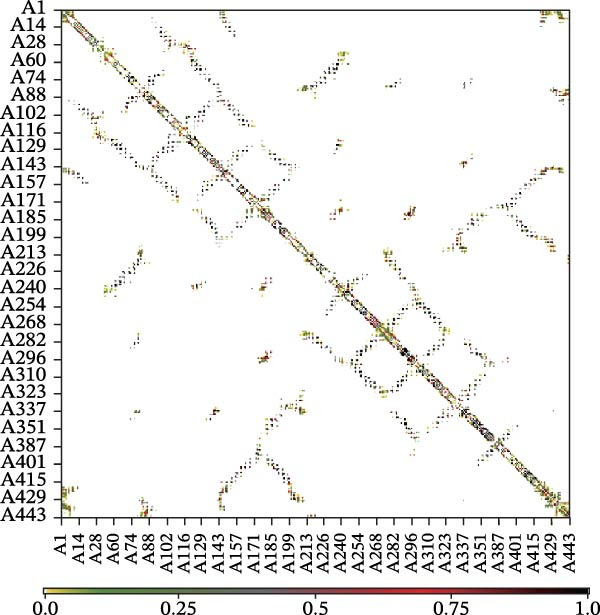
(I)

**Figure:**
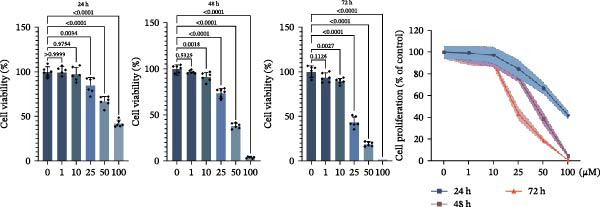
(J)

**Figure:**
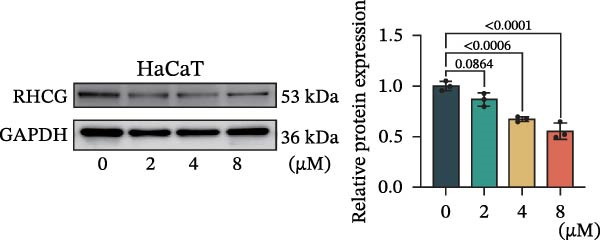
(K)

**Figure:**
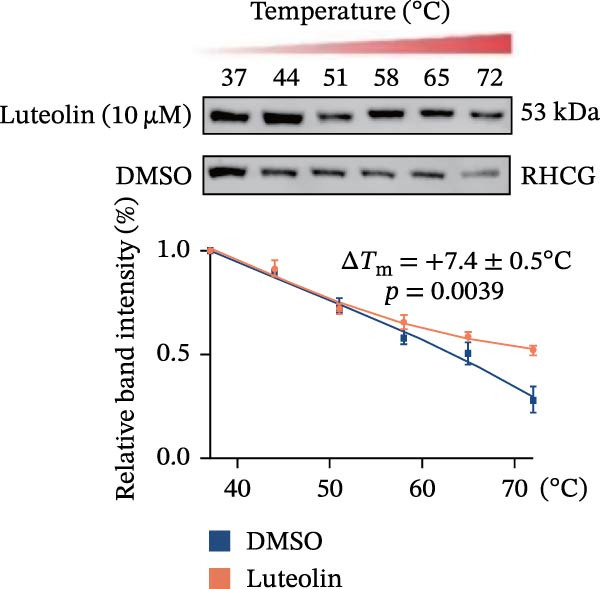
(L)

To further assess complex stability and global motions, we analyzed the docked structures using the iMODS, which performs normal mode analysis (NMA). NMA captures low‐frequency modes that reflect slow and large‐scale conformational changes in the complex. The RHCG–luteolin NMA outputs are summarized in Figure 2C–I. Deformability and B‐factor plots were used to profile residue mobility, where prominent peaks indicate regions with higher predicted flexibility. The B‐factor plot also allows a qualitative comparison between NMA–predicted mobility and the crystallographic B‐factors reported in the PDB (Figure 2C–I).

Subsequent experimental validation of the RHCG–luteolin binding interaction was conducted. HaCaT cell viability in response to luteolin was evaluated using CCK‐8 assays. Cells were exposed to luteolin concentrations ranging from 0 to 100 μM, with viability assessed at multiple time points. Luteolin significantly suppressed proliferation in dose‐ and time‐dependent manners (Figure 2J). Of note, 10 μM luteolin treatment exhibited no significant viability effects at 24 h; this dose was consequently selected for subsequent mechanistic investigations. Western blot analysis showed a dose‐dependent reduction in RHCG protein levels following 24 h luteolin treatment (Figure 2K). To further confirm target engagement, CETSA were performed in vitro. CETSA analysis demonstrated that 10 μM luteolin enhanced RHCG thermal stability in HaCaT cells, consistent with direct binding (Figure 2L). The uncropped blots for Supporting Information 3: Figure 2K,L are provided in the “Uncut blot” supporting information.

### 3.3. Luteolin Attenuates KC Inflammation via RHCG Downregulation, Leading to the Prevention of DC Activation

Our team’s previous research has shown that upregulation of RHCG promotes inflammation levels in KCs and enhances the CXCL14–CXCR4 signal, thereby leading to DC activation [26]. Since we have confirmed that luteolin can target RHCG, we further studied the effects of luteolin on KCs and DCs. We first downloaded a scRNA‐seq dataset, GSE151177, from the GEO database. After batch correction and unsupervised clustering, we annotated 24 cell lineages based on established marker genes (Figure 3A). These included granular KCs, basal KCs, mature regulatory DCs (mregDCs), conventional DCs (cDCs), CD4 + T cells, naïve CD4 + T cells, regulatory T cells (Treg cells), GZMB+CD8+ T cells (granzyme B‐positive CD8+ T cells), memory CD8+ T cells (memory CD8+ T cells), natural killer cells (NK cells), B cells, monocytes, macrophages, M1 macrophages, venous endothelial cells (venous ECs), lymphatic endothelial cells (lymphatic ECs), tip ECs, and CCL19+APOE+fibroblasts. The proportion of each cell lineage varies greatly between normal and psoriasis tissues (Figure 3B). According to the AUCell scoring method, we mapped the acquired target expression profiles of luteolin onto the scRNA‐seq dataset. As illustrated in Figure 3C, the luteolin‐related targets were mainly enriched in psoriasis than normal skins. More importantly, KC, mregDC, and monocyte are the main target cells of luteolin (Figure 3D).

Figure 3Luteolin improves keratinocytes (KCs) inflammation and inhibits dendritic cells (DCs) activation through RHCG. (A) Uniform manifold approximation and projection (UMAP) projection of 28,880 cells from scRNA‐seq data across 23 samples (six normal skin samples and 17 psoriasis samples), identifying 24 cell lineages: granular keratinocyte, basal keratinocyte, mregDC, CD4 T cell, NK cell, venous EC, lymphatic EC, cycling keratinocyte, spinous keratinocyte, melanocyte, macrophage, KRT14 basal cell, secretory cell, GZMB CD8 T cell, monocyte, tip EC, B cell, M1 macrophage, cDC, CCL19+APOE + fibroblast, Treg cell, naive CD4 T cell, memory CD8 T cell, and endothelial cell. (B) Stacked bar plot depicting the proportion of each cell lineage across tissue types. (C) Violin plots showing the differences in AUCell scores of potential target genes of luteolin between normal and psoriasis tissues. (D) UMAP projection of AUCell scores of potential target genes of luteolin. Brighter colors indicate stronger targeting intensity in specific cell lineages. (E, F) UMAP projection of AUCell scores of potential target genes of luteolin (E) and glycolysis (F) in 13,739 keratinocytes. (G) UMAP projection of *RHCG*, *KRT16*, *KRT17*, and *S100A12* in 13,739 keratinocytes. (H) Western blot analysis of HK2, KRT1, KRT16, and S100A12 expression in HaCaT cells in different treatments. (I) Elisa of intracellular lactate level in each group. (J) Schematic of the noncontact transwell coculture system (0.4 μm pore size) used to assess KC–DC communication. HaCaT cells were transfected (NC or oe‐RHCG), stimulated with M5 for 24 h, then treated with luteolin (10 μM) or MTX (50 ng/mL) for 24 h. After treatment, HaCaT cells were washed and replaced with fresh drug‐free medium and seeded in the upper inserts (1 × 10^5^ cells/well), while DCs were seeded in the lower chamber (5 × 10^4^ cells/well). Cells were cocultured for 24 h, followed by collection of DCs and supernatants for downstream analyses. (K) Western blot analysis of LAMP3, CD80, and CD86 expression in DCs after KC–DC transwell coculture under the indicated conditions. (L, M) ELISA quantification of IL‐23, IL‐6 (L) and CXCL14 (M) levels in the coculture supernatant from each group. Statistical analysis: All in vitro experiments were independently repeated three times. Data are presented as mean ± SD, and statistical significance was assessed using one‐way ANOVA with Tukey’s post hoc test. Exact *p* values (or significance levels) are indicated on the figure.

**Figure:**
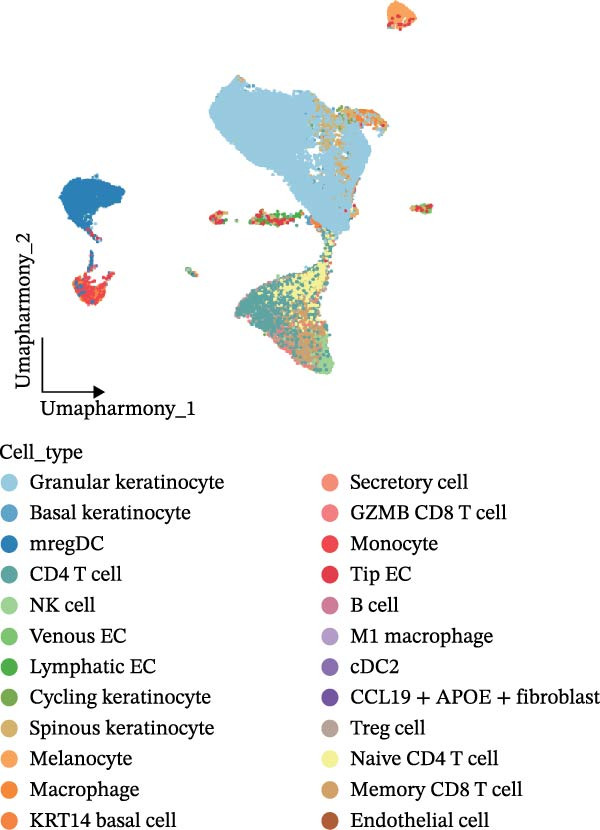
(A)

**Figure:**
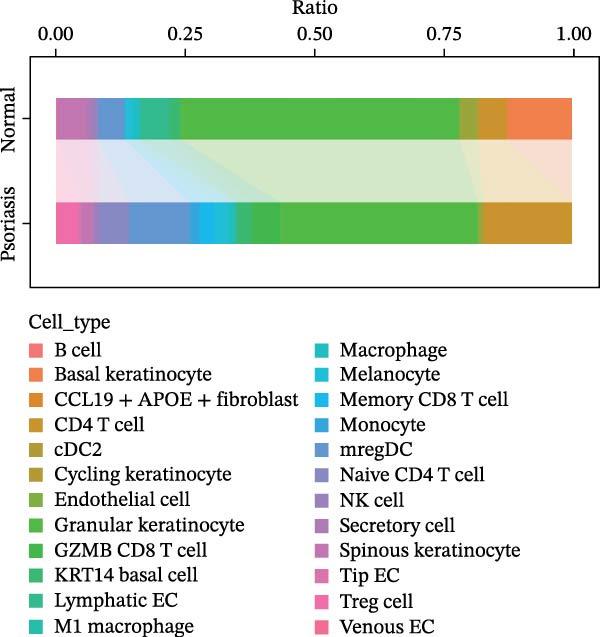
(B)

**Figure:**
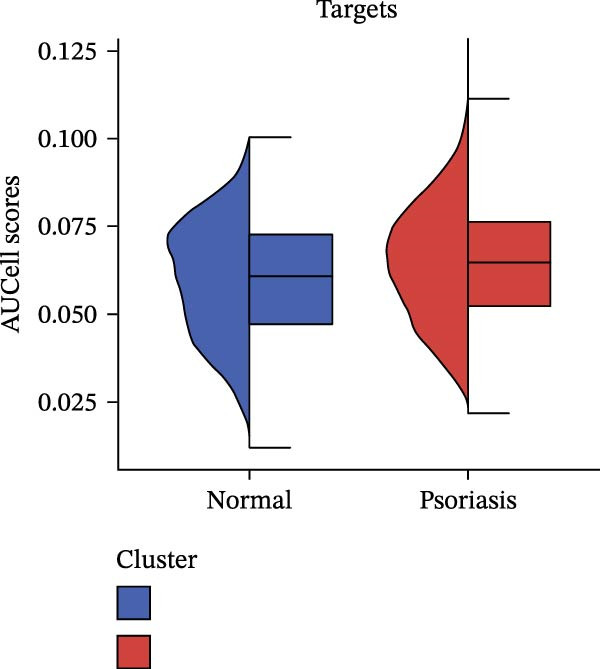
(C)

**Figure:**
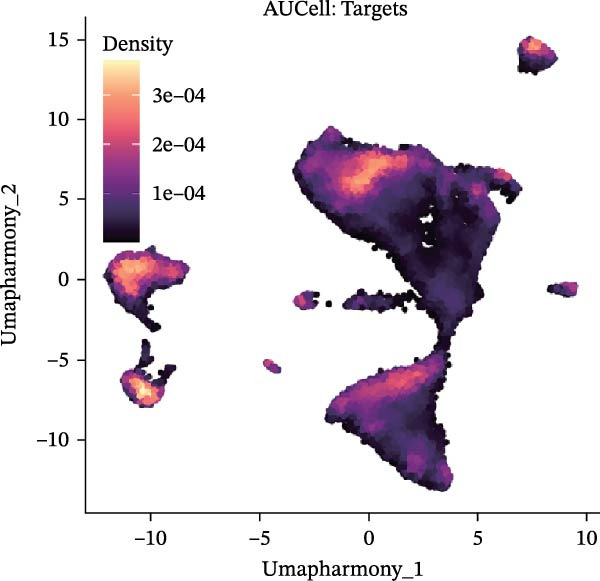
(D)

**Figure:**
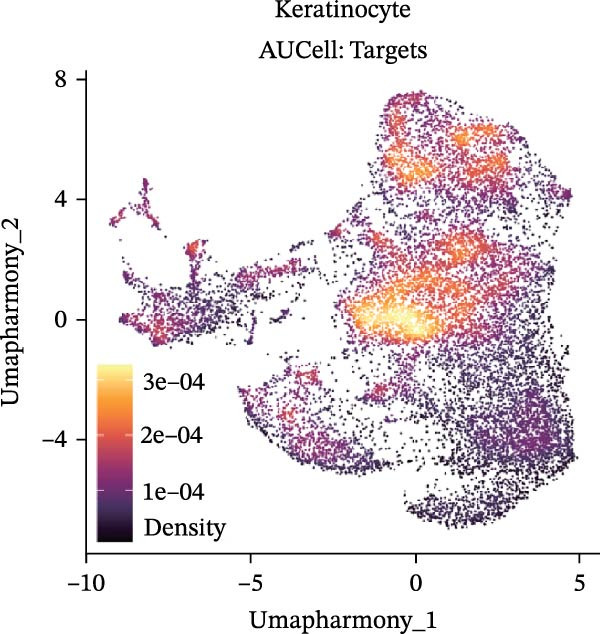
(E)

**Figure:**
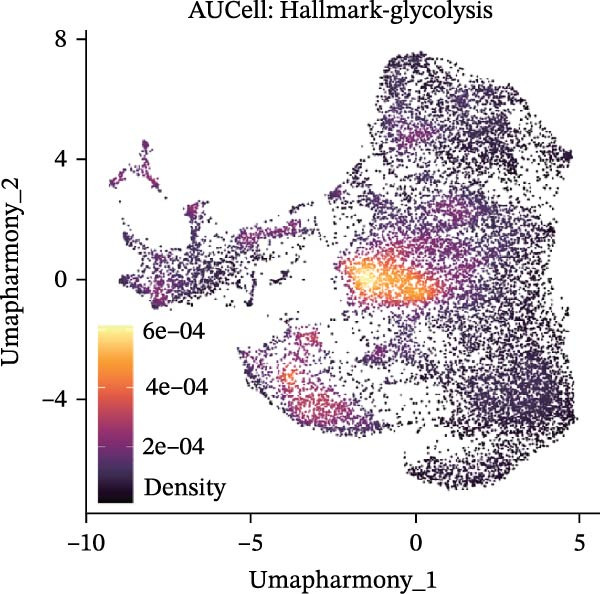
(F)

**Figure:**
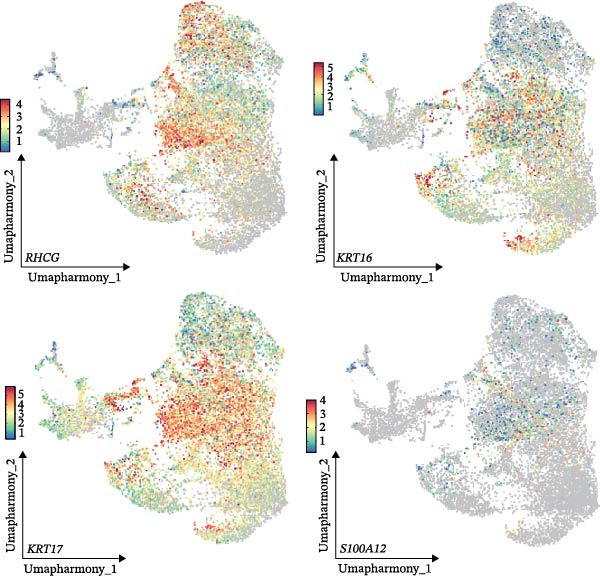
(G)

**Figure:**
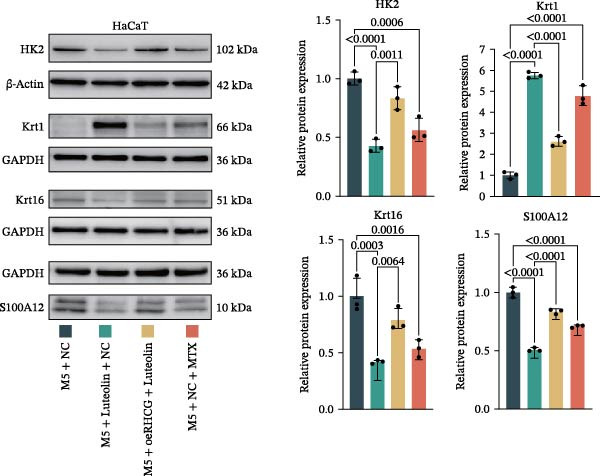
(H)

**Figure:**
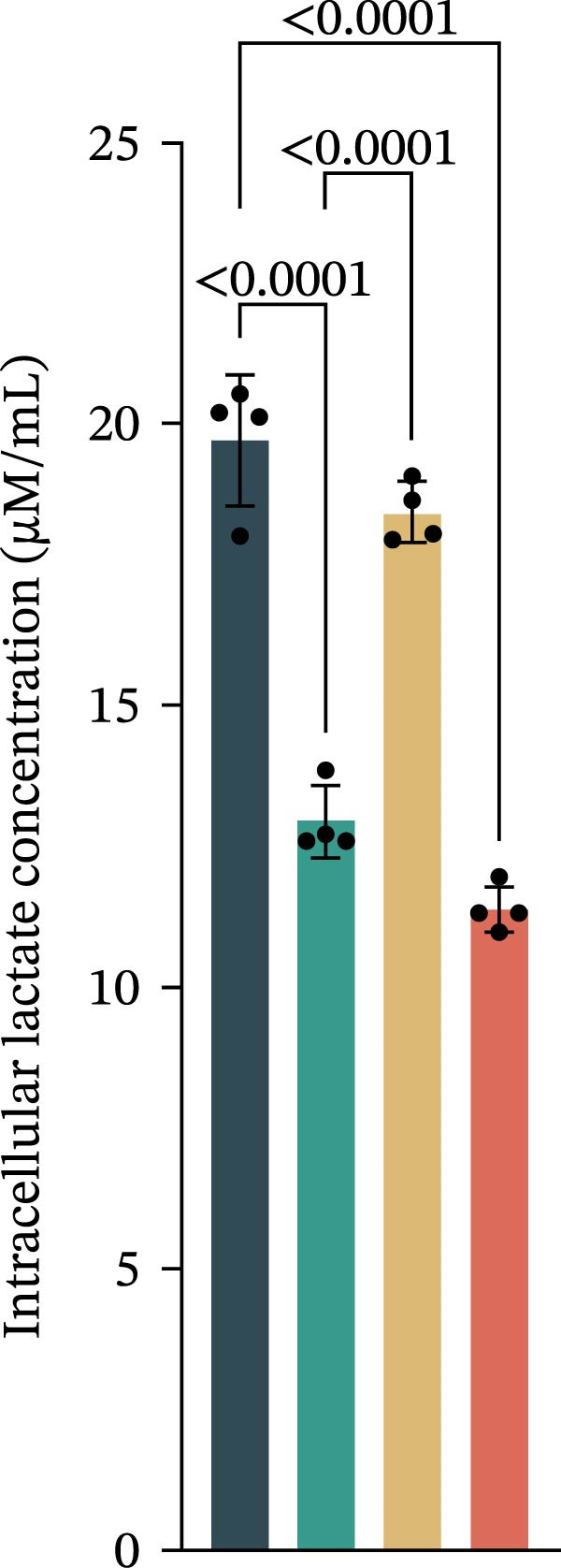
(I)

**Figure:**
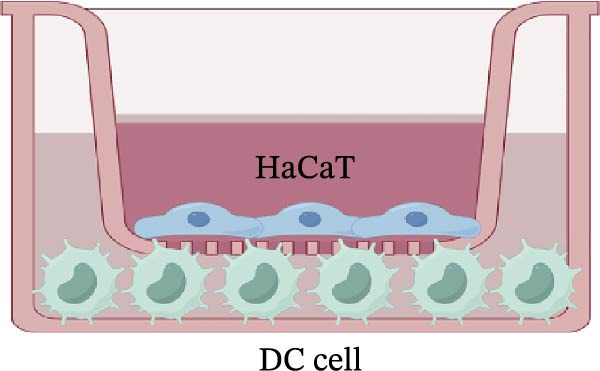
(J)

**Figure:**
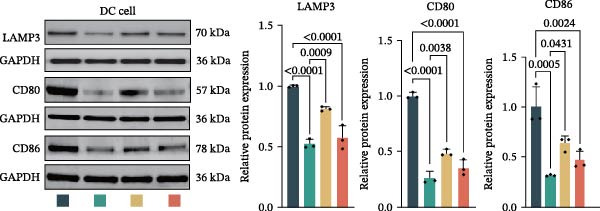
(K)

**Figure:**
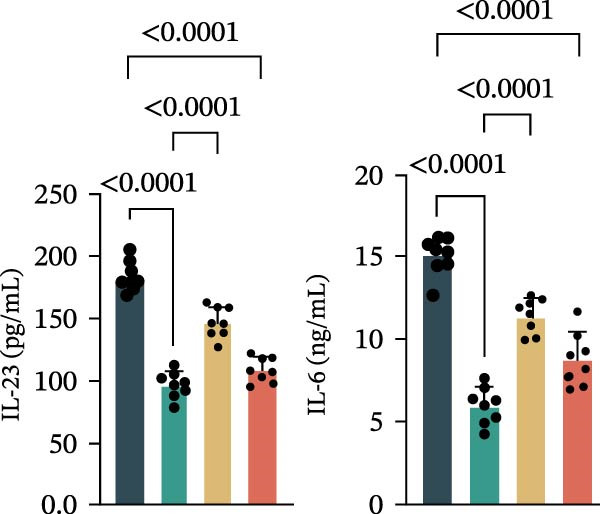
(L)

**Figure:**
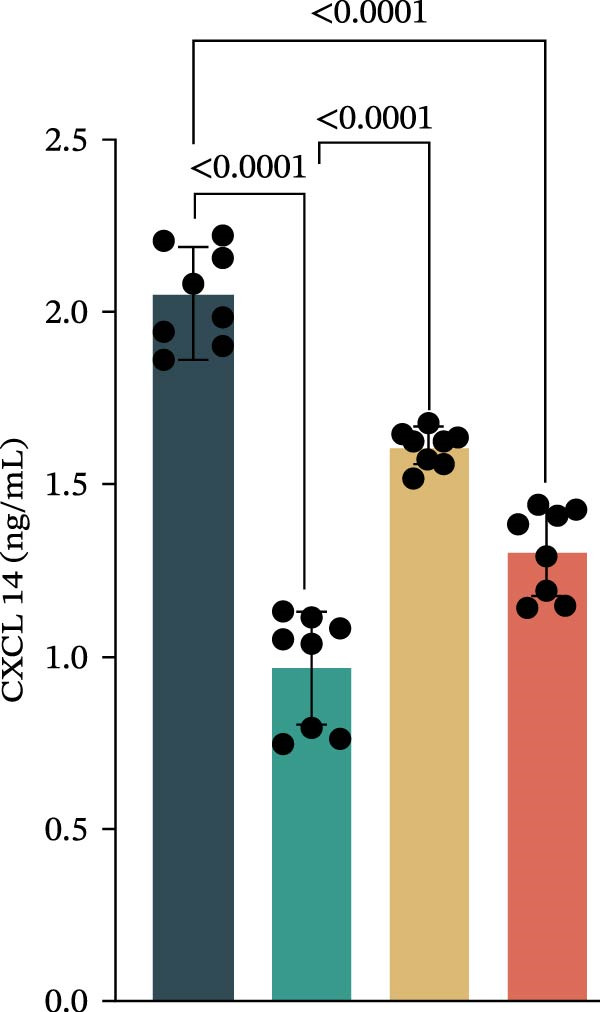
(M)

Within the intricate inflammatory network characteristic of psoriasis, KCs are generally recognized to occupy a central and pivotal position [59]. We performed clustering analysis of 13,739 KCs and revealed six prominent cell subgroups (data not shown). We further observed that KCs potentially targeted by luteolin exhibited enhanced glycolysis levels, accompanied by high expression of *RHCG*, *KRT16*, *KRT17*, and *S100A12* (Figure 3E–G). These results suggested that luteolin may have a potential regulatory effect on inflammation and glycolysis of KCs. To validate this hypothesis, we conducted in vitro experiments. As shown in Figure 3H, compared with the model group, luteolin significantly downregulated the expression of S100A12, KRT16, and HK2 (a key enzyme of glycolysis), but restored the expression of KRT1, with effects similar to those of MTX treatment. The uncropped blots for Supporting Information 3: Figure 3H are provided in the “Uncut blot” supporting information. However, these therapeutic effects were rescued by oe‐RHCG. In addition, luteolin was observed to downregulate lactate levels via RHCG in cell supernatants (Figure 3I).

Previous work from our team demonstrated that RHCG promotes KC–derived CXCL14 signaling to DCs, enhancing their maturation [26]. To determine whether luteolin modulates DC activation, we employed a KC–DC coculture model (Figure 3J). Luteolin treatment significantly attenuated DC activation, as demonstrated by reduced expression of the maturation marker LAMP3, diminished surface levels of the costimulatory molecules CD80 and CD86 (Figure 3K), and decreased secretion of the pro‐inflammatory cytokines IL‐23 and IL‐6 (Figure 3L). The uncropped blots for Supporting Information 3: Figure 3K are provided in the “Uncut blot” supporting information. In parallel, luteolin also reduced the level of CXCL14 in the coculture supernatant. Consistent with a key role for RHCG in this regulatory axis, the suppressive effects of luteolin on these DC activation markers, cytokine secretion, and CXCL14 release were largely abolished when RHCG was overexpressed in KCs (oe‐RHCG; Figure 3M). These findings collectively indicate that luteolin disrupts KC–DC communication.

### 3.4. Luteolin Has a Potential Regulatory Effect on DCs‐Dominant Spatial Domains

Psoriasis pathogenesis involves complex communication networks between KCs, DCs, and other immune cells, spatially organized into distinct microdomains (cellular niches) [60]. These specialized niches, defined by unique cellular compositions and molecular signatures, engage in critical cross talk through chemokine gradients and receptor–ligand interactions. This spatially coordinated inter‐niche communication orchestrates inflammatory cascades, drives KC dysregulation, and sustains disease progression throughout the psoriatic lesion [61]. Recent spatial analyses highlight these dynamic niches as fundamental organizational and functional units in the disease process [62, 63].

We obtained STs data for four psoriasis samples (VISDS001018, VISDS001019, VISDS001020, and VISDS001021; Figure 4A) from the CROST portal. Because Visium spots represent mixtures of multiple cells, we applied BayesSpace to perform spatial clustering and used CROST‐provided deconvolution results to estimate cell‐type proportions. We then integrated these outputs with SpaTopic to group spots into cellular topics (spatial domains). As shown in Figure 4B, this approach yielded six domains in VISDS001018, nine domains in VISDS001019, five domains in VISDS001020, and seven domains in VISDS001021, outlining distinct histology‐associated regions across the psoriatic sections.

Figure 4Luteolin has potential regulatory effects on the DC–dominant spatial domains. (A) Hematoxylin/eosin (HE) staining of four psoriasis samples (VISDS001018–VISDS001021) from the CROST database. Scale Bar, 500 μm. (B) The integration of the “BayesSpace” spatial clustering method and the “SpaTopic” pipeline allowed the categorization of spatial spots into six topics in VISDS001018, nine topics in VISDS001019, five topics in VISDS001020, and eight topics in VISDS001021. (C) Luteolin‐related targets were mapped onto the spatial topics, revealing preferential targeting of specific domains. Luteolin predominantly targeted Topic2/4 in VISDS001018, Topic1/5/6 in VISDS001019, Topic2/4 in VISDS001020, and Topic4/6/7 in VISDS001021, suggesting selective interaction with regions enriched in specific cell types. (D) Half‐violin plots showing the cellular composition of identified spatial domains, with unique mixtures of different cell types across the different regions. (E) Spatial networks of four psoriasis samples. The networks highlight strong connectivity among luteolin‐targeting, DC–dominated topics within each sample, reflecting their spatial proximity. (F) Heatmaps depicting neighborhood enrichment analysis of luteolin‐targeting spatial domains in four psoriasis samples. Red represents high *Z*‐scores, and blue represents low *Z*‐scores, demonstrating that DC–dominated domains targeted by luteolin are spatially close within each sample, indicating strong colocalization.

**Figure:**
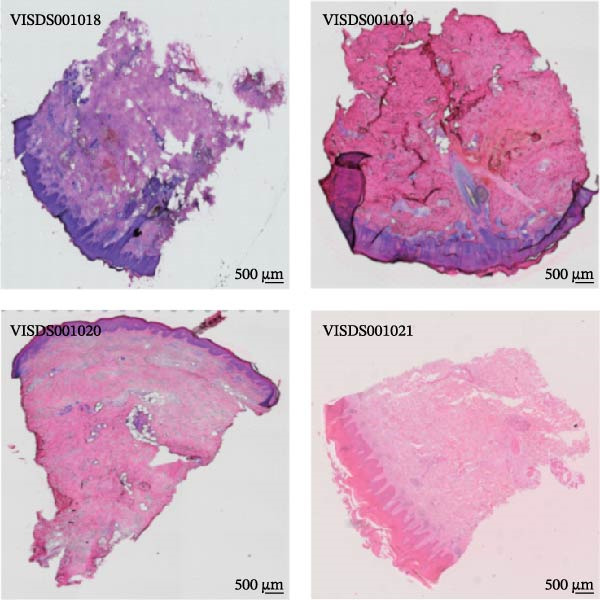
(A)

**Figure:**
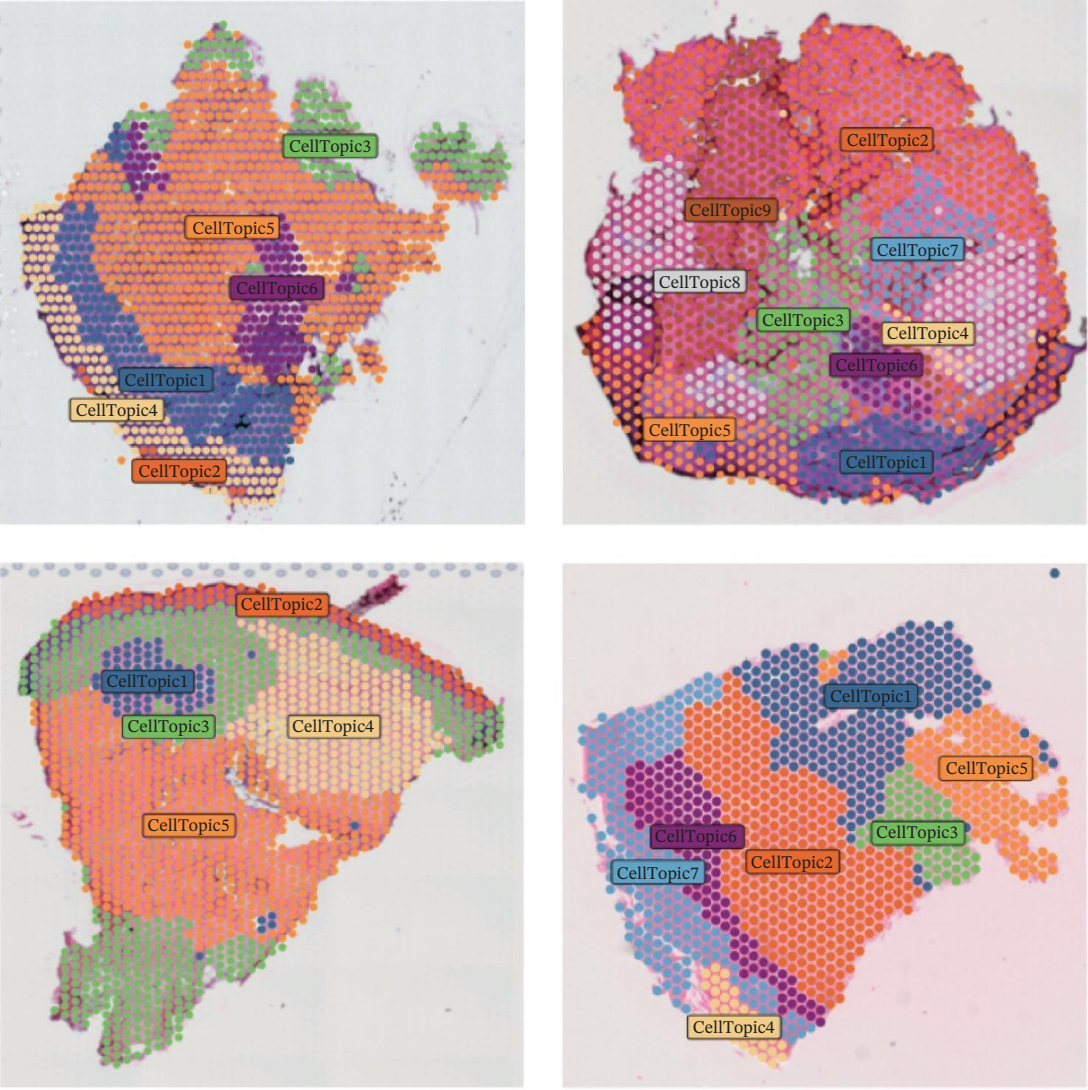
(B)

**Figure:**
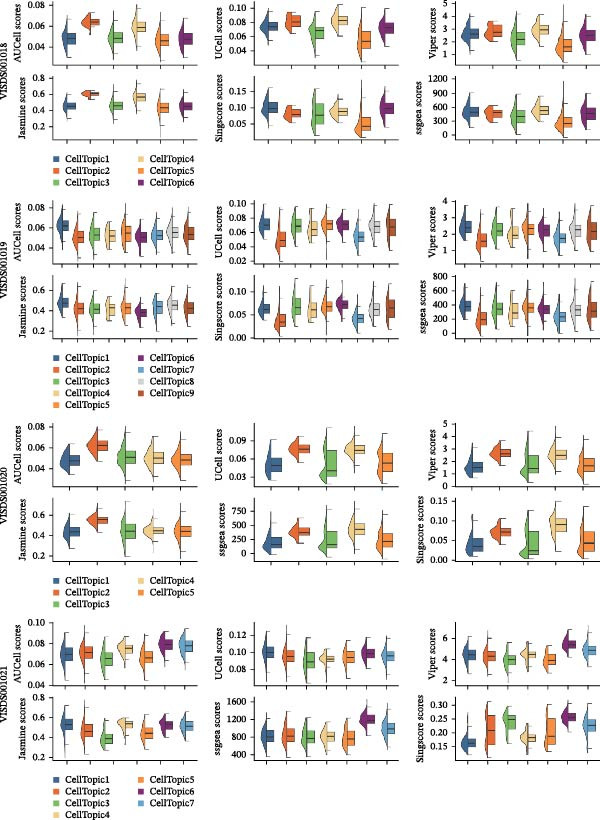
(C)

**Figure:**
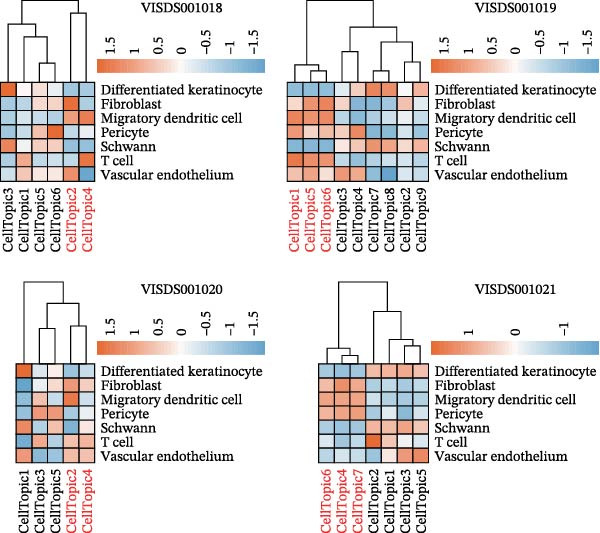
(D)

**Figure:**
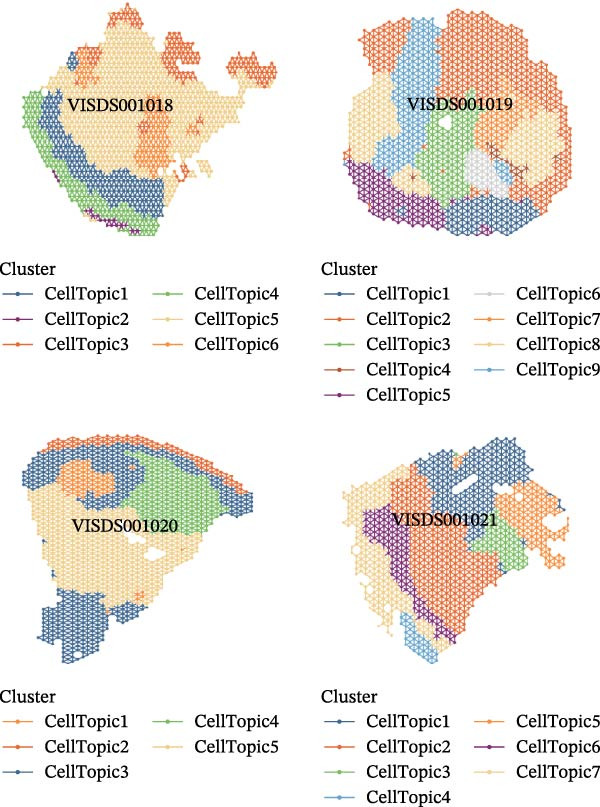
(E)

**Figure:**
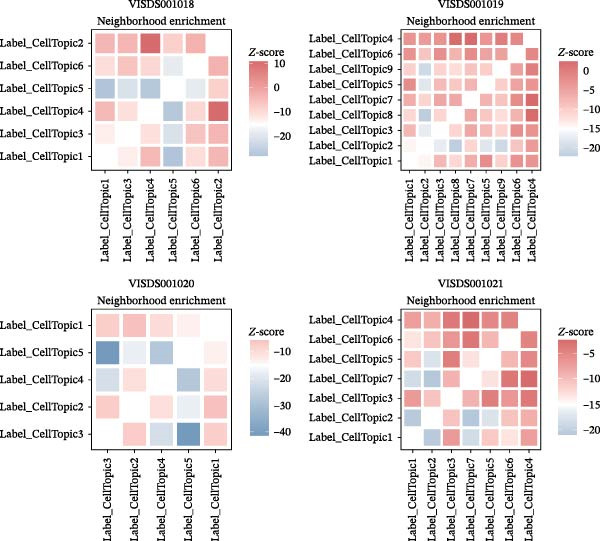
(F)

We then mapped putative luteolin targets onto this spatial architecture to identify its preferential targeting domains. Intriguingly, luteolin exhibited domain‐specific targeting patterns across samples: Topics 2/4 (VISDS001018), Topics 1/5/6 (VISDS001019), Topics 2/4 (VISDS001020), and Topics 6/7 (VISDS001021; Figure 4C). Deconvolution analysis of these luteolin‐targeting domains revealed DCs as a consistently dominant cellular component (Figure 4D). Notably, all targeted domains displayed minimal infiltration by differentiated KCs and clustered transcriptionally within each sample, suggesting shared molecular characteristics.

To further characterize these domains, we assessed their spatial colocalization patterns using neighborhood enrichment analysis. This revealed significant spatial proximity among luteolin‐targeting, DC–enriched domains, implying potential functional correlations (Figure 4E,F). Collectively, these findings suggest luteolin may exert its therapeutic effects by selectively targeting spatially defined niches with specific cellular and molecular features.

### 3.5. DESMOSOME and GAP Signaling Are Key Signals in the Luteolin Targeting Domains

Because spatial domains can represent organized functional zones within the tissue microenvironment, examining how domains communicate helps clarify whether luteolin acts mainly in specific regions or more broadly across the tissue. We, therefore, used CellChat to estimate communication probabilities both within domains and between domains. Luteolin‐targeted domains showed frequent within‐domain signaling and also strong interactions with other domains (Figure 5A). We then summarized signaling pathways in a domain‐resolved manner and classified them as intradomain or interdomain communication. Across pathways associated with luteolin‐targeted domains, DESMOSOME and GAP signaling stood out, showing high communication probabilities in both within‐domain and between‐domain settings (Figure 5B). The corresponding spatial flow patterns are shown in Figure 5C.

Figure 5Luteolin‐targeting domains have activated DESMOSOME and GAP signaling. (A) Heatmaps depict the number of interactions (A1) and interaction strength (A2) among cellular topics across four psoriasis samples. Color‐coded edges indicate signaling strength, with red representing higher communication activity. An adjacent bar graph quantifies the signaling intensity, where bar height corresponds to the strength of communication within or between regions. (B) Bubble plots showing intraspatial (B1) and interspatial (B2) domain communication across four psoriasis samples. Topics and signaling pathways are plotted along the *x*‐ and *y*‐axes, respectively, with DESMOSOME and GAP signaling pathways highlighted in red. Bubble color indicates communication probability (red: high; pink: low), and bubble size reflects statistical significance (larger bubbles correspond to smaller *p*‐values). The plots reveal significant communication both within and between luteolin‐targeted domains, primarily mediated by DESMOSOME and GAP signaling. (C) Spatial diagrams illustrate the intraspatial communication patterns of the DEMOSOME and GAP signaling pathways across four psoriasis samples. (D) Bar plots display network centrality scores (ranging from 0 to 1, white to red) for DEMOSOME and GAP signaling pathways. Luteolin‐targeted topics are identified as key senders and influencers, suggesting a potential mechanistic link between luteolin and these signaling processes. (E) Contribution analysis of ligand–receptor pairs to the DEMOSOME pathway in four psoriasis samples, ranked from highest to lowest score. Ligand–receptor information of the GAP pathway is omitted due to relative lack of data.

**Figure:**
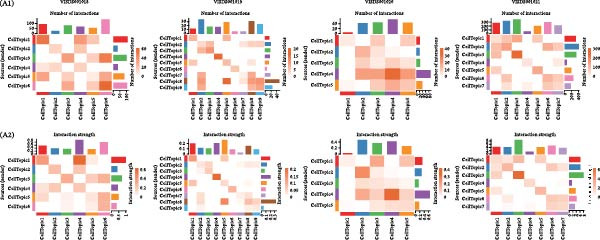
(A)

**Figure:**
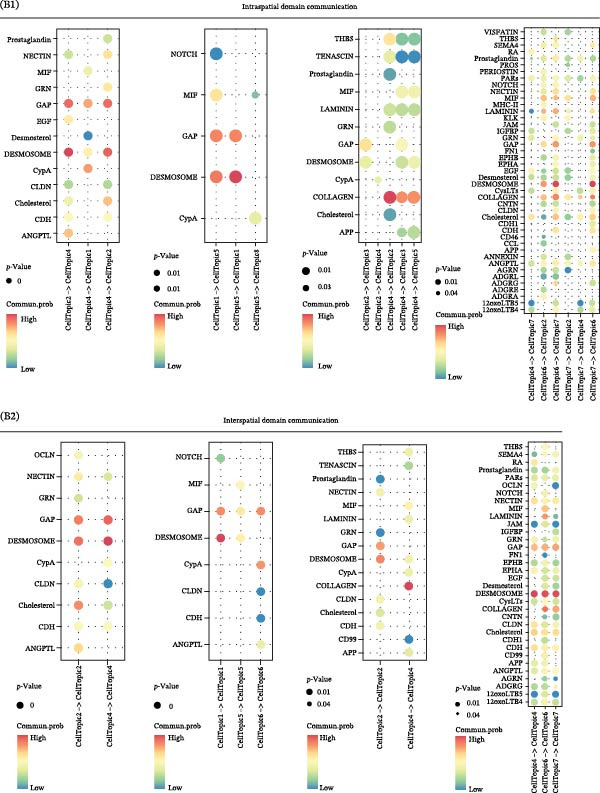
(B)

**Figure:**
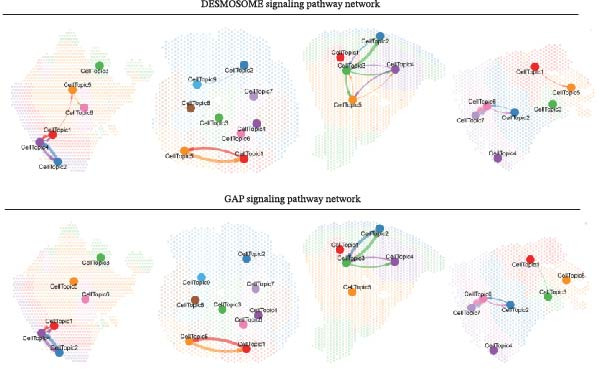
(C)

**Figure:**
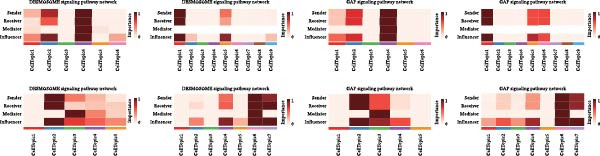
(D)

**Figure:**
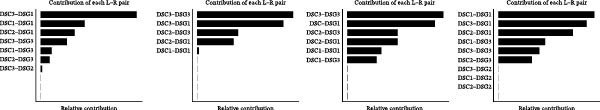
(E)

To delineate the key roles of these pathways within the luteolin‐targeting communication network, we calculated network centrality scores for each domain. This analysis revealed that luteolin‐targeting domains consistently functioned as the primary senders and influencers of DESMOSOME and GAP signaling across all four psoriasis samples (Figure 5D), suggesting a potential link between these pathways and luteolin intervention.

Focusing on these two key luteolin‐associated signals, we subsequently identified ligands and receptors common to both pathways to pinpoint luteolin’s precise protein targets. We assessed the contribution of each ligand‐receptor pair to the overall signaling strength. Figure 5E showed the predominant ligands and receptors in DESMOSOME signaling pathways. GAP signaling was excluded from further analysis owing to limited available data on its cognate ligands and receptors. Desmosomes, which are essential for epidermal barrier integrity, are significantly downregulated in psoriatic lesions. This downregulation contributes to compromised cell–cell adhesion and impaired barrier function, thereby facilitating inflammatory cell infiltration and disease pathogenesis.

### 3.6. RHCG Is Associated With Desmosomes in Psoriasis

Spatial analysis suggested that DESMOSOME signaling may function as a downstream pathway of luteolin intervention. We, therefore, investigated the relationship between DESMOSOME signaling and RHCG. First, we assessed this potential association at the transcriptomic level using three publicly available GEO datasets (GSE13355, GSE14905, and GSE30999). PCA revealed distinct gene expression patterns between nonlesional and lesional samples, a consistency observed across all bulk‐seq datasets (Figure 6A). Notably, ligands and receptors within the DESMOSOME pathway exhibited significant differential expression between these groups (Figure 6B). This finding was further corroborated by scRNA‐seq data, which demonstrated upregulated transcription of *DSC2* and *DSG3* in psoriatic tissues (Figure 6C). We subsequently evaluated the correlation between RHCG and DESMOSOME signaling components in both normal and diseased tissues (Figure 6D). *RHCG* expression displayed tissue‐specific correlations with individual DESMOSOME members. The expression profiles of desmosomal cadherins in skin disorders exhibit marked heterogeneity across protein subtypes and epidermal layers: DSG1 demonstrates paradoxical upregulation at the mRNA level in lesional epidermis despite protein depletion in the upper strata, while DSC1 shows transcriptional suppression, contrasting with DSC3’s predominant posttranslational degradation via protease‐mediated cleavage in the basal/spinous layers [64–66]. This compartmentalized dysregulation—spanning transcriptional, translational, and posttranslational tiers—collectively disrupts epidermal cohesion and mirrors the pathophysiological stratification seen in psoriatic plaques (differentiation defects in superficial layers vs. hyperproliferation in deeper compartments). Importantly, a positive correlation between *RHCG* and *DSC2* was observed in both scRNA‐seq and bulk‐seq data. Consistent with this, the mRNA expression level of *DSC2* was significantly elevated in psoriatic tissues (Figure 6D,E). However, these findings appear to contradict the well‐established downregulation of desmosomal proteins in psoriasis [67]. To resolve this apparent discrepancy—and considering the frequent discordance between transcriptional and translational regulation—we performed mIF staining on 30 normal and 30 psoriatic skin samples. As demonstrated in Figure 6F, DSC2 protein expression was significantly reduced in lesional tissues compared to normal controls. Notably, while DSC2 and RHCG protein levels showed a weak positive correlation in normal skin, a strong negative correlation was observed in psoriatic lesions (Figure 6G). To further investigate the possible molecular mechanisms underlying these observations, we analyzed the correlation between *RHCG* and *MMP9*, *KLK5*, and *KLK7* expression in both normal and psoriatic skin samples. KLK5 and KLK7 are serine proteases that are upregulated in inflammatory skin conditions like psoriasis and are known to degrade desmosomal cadherins, contributing to epidermal barrier dysfunction and stratum corneum integrity disruption [68, 69]. MMP9, a matrix metalloproteinase, is also elevated in psoriatic skin and is implicated in the breakdown of extracellular matrix components, exacerbating epidermal inflammation [70]. The chord diagrams in Figure 6H show that in psoriatic tissues, RHCG exhibits varying degrees of positive correlation with KLK5, KLK7, and MMP9, whereas no significant correlation is observed in normal skin. These results suggest that RHCG plays a role in the regulation of desmosomal components at the protein level through protein degradation, potentially influencing desmosomal integrity and contributing to epidermal dysfunction in psoriasis.

Figure 6RHCG is related to DESMOSOME signaling. (A) Principal component analysis (PCA) result visualization based on tissue nature in GSE14905, GSE30999, and GSE13355. There are significant differences in transcriptional characteristics between normal samples and psoriasis samples. (B) The differences of the ligands and receptors in DEMOSOME signaling between normal and lesional tissues in the three independent GEO datasets (GSE14905, GSE30999, and GSE13555). (C) The differences of the ligands and receptors in DEMOSOME signaling between normal and lesional tissues in the scRNA dataset GSE151177. (D) The chord diagrams show the correlation between *RHCG* and the ligands and receptors in DEMOSOME signaling in three bulk datasets. (E) The scatter plots show the correlation between *RHCG* and the ligands and receptors in DEMOSOME signaling in the scRNA dataset. (F) Representative HE and multiplex immunofluorescence (mIF) staining of human psoriasis and normal skin tissue. KRT16 (gold), RHCG (red), DSC2 (green), and DAPI (blue) in individual and merged channels are shown. Scale bar, 50 μm. Experiment was performed in 30 independent patients (30 psoriasis samples and 30 adjacent normal controls). (G) The Pearson correlation coefficient between RHCG and DSC2 was calculated in psoriasis (*R* = 0.226) and normal (*R* = −0.400) skin, respectively. N, normal tissues; P, psoriasis tissues. (H) The chord diagrams show the correlation between *RHCG* and *KLK5*, *KLK7*, and *MMP9* in normal (N) and psoriatic (P) skin samples from three independent GEO datasets (GSE14905, GSE30999, and GSE13555). In psoriatic tissues, *RHCG* exhibits varying degrees of positive correlation with *KLK5*, *KLK7*, and *MMP9*, whereas no significant correlation is observed in normal skin.

**Figure:**
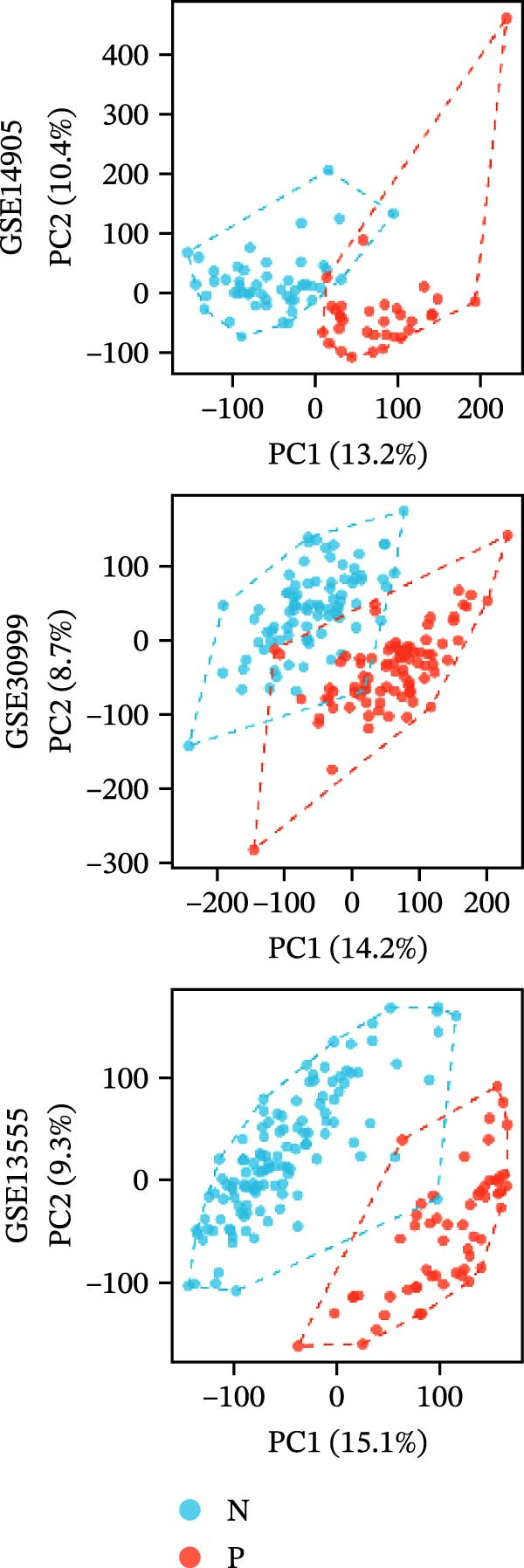
(A)

**Figure:**
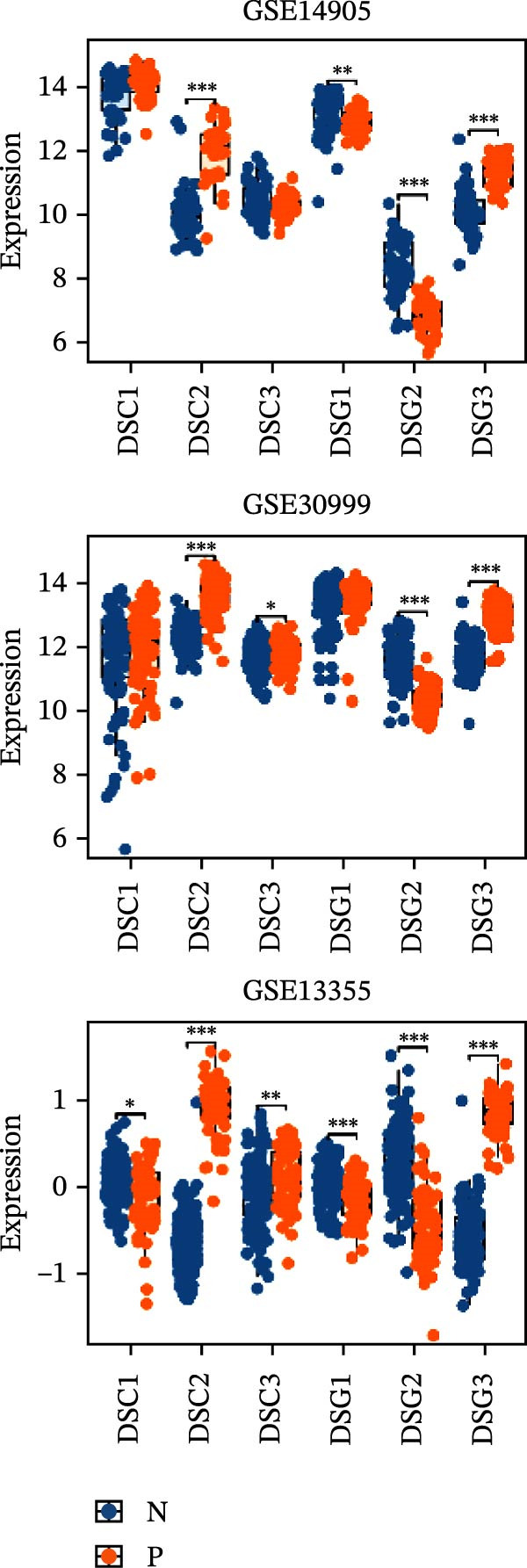
(B)

**Figure:**
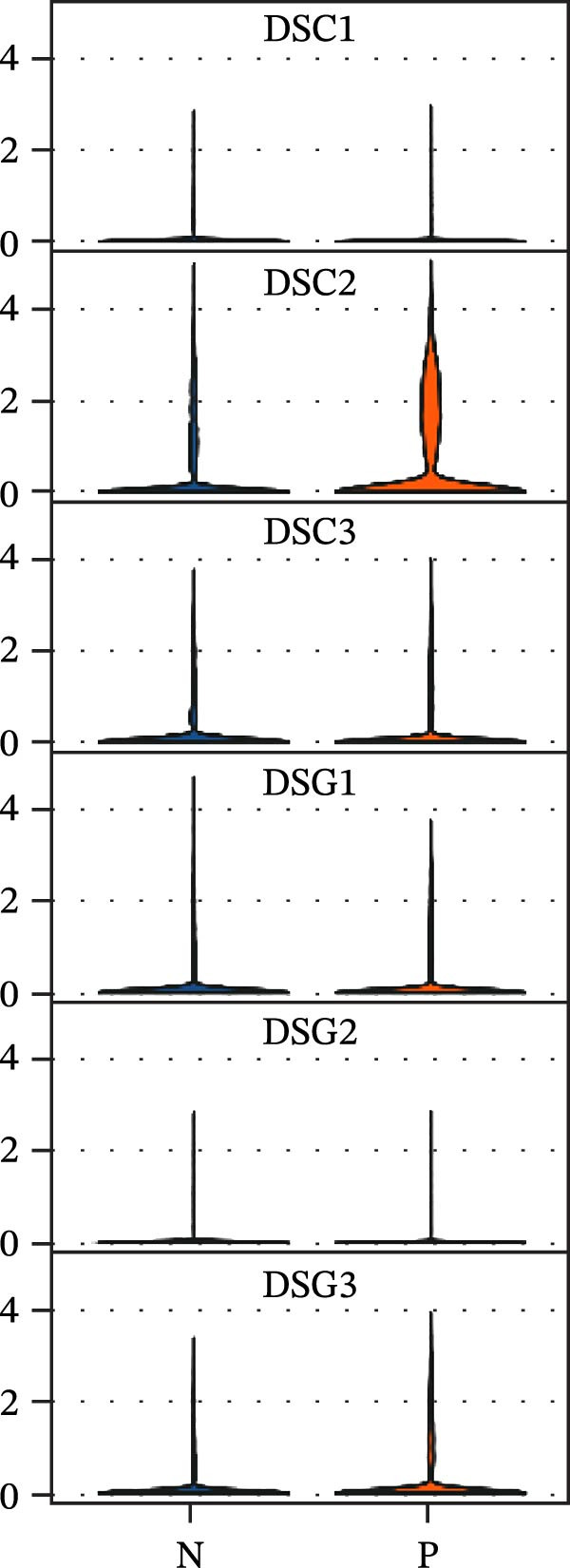
(C)

**Figure:**
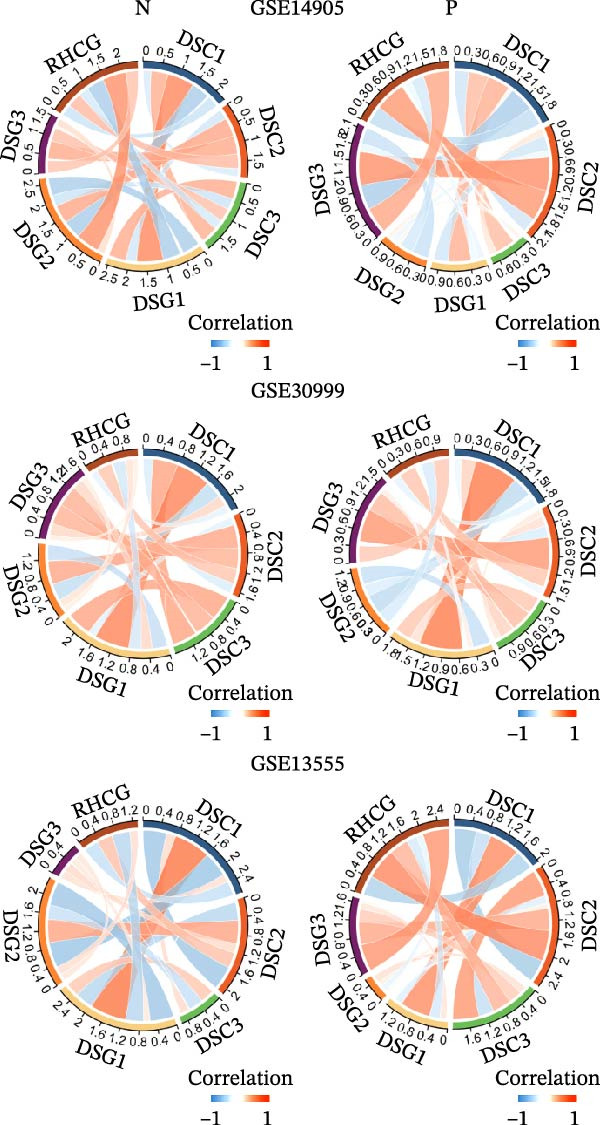
(D)

**Figure:**
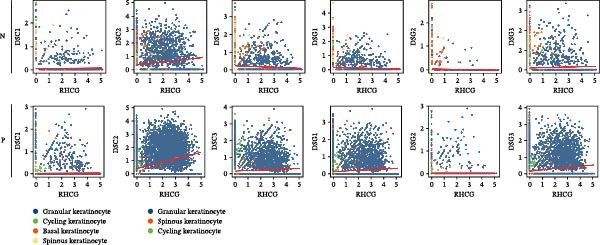
(E)

**Figure:**
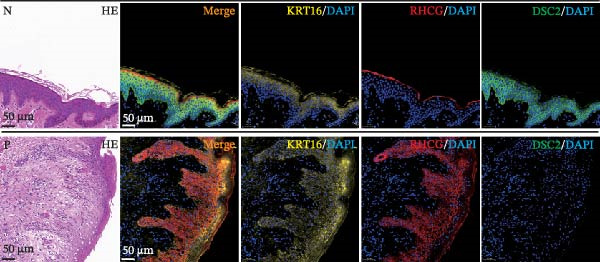
(F)

**Figure:**
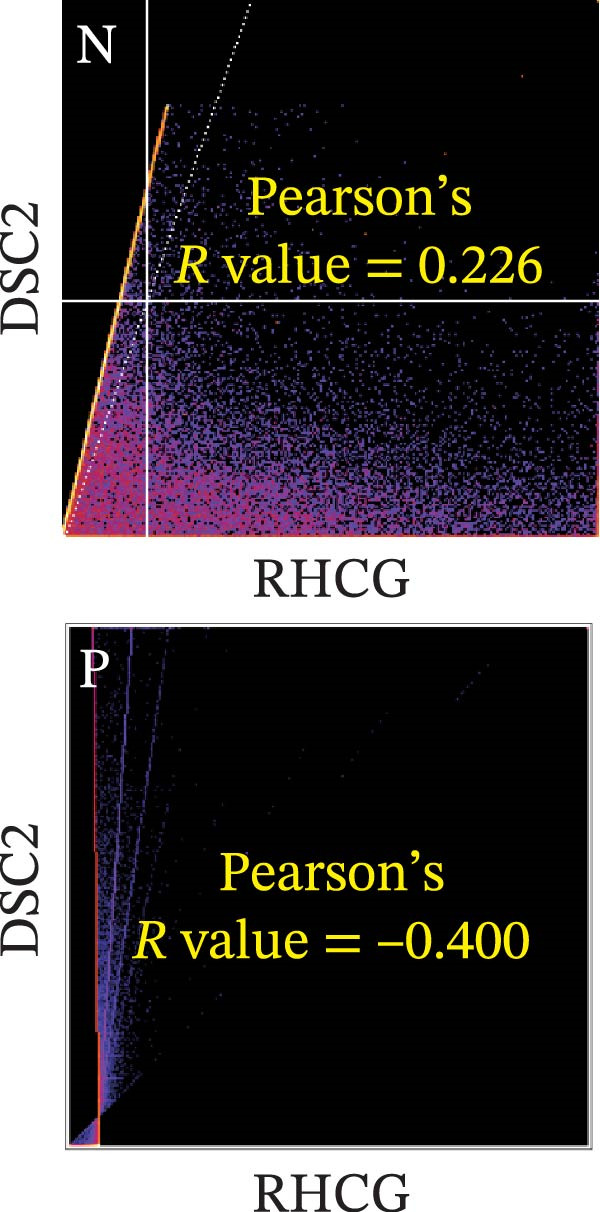
(G)

**Figure:**
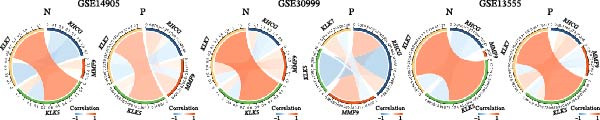
(H)

### 3.7. Luteolin Regulates DCs‐Dominant Spatial Domains and Recovers Desmosome Protein Expression

As illustrated in Figure 7A, luteolin‐targeting regions exhibited elevated densities of DCs and reduced levels of differentiated KCs, along with heightened expression of *RHCG*, *KRT16*, and *KRT17*. Next, we conducted in vivo experiments to validate the predictions derived from spatial analysis. Throughout the experimental period, all mice maintained normal physiological status and health parameters, including stable food and water intake as well as regular respiratory patterns.

Figure 7In vivo validation of luteolin treatment for psoriasis and intervention in DC–dominant domains. (A) Spatial diagrams illustrate the colocalization of *RHCG*, *KRT16*, and *KRT17* in luteolin‐targeting domains in four psoriasis samples, accompanied by high levels of DCs and low levels of differentiated KCs. Magnified views are provided to highlight representative regions. (B) All images of dorsal skin from imiquimod (IMQ; model) group, control group, and treatment groups (MTX for Methotrexate and luteolin; *n* = 6). Luteolin treatment markedly reduced erythema, scaling, and thickening of psoriatic lesions compared to the IMQ group. (C) Erythema, scale, infiltrate, and severity score across different groups. Luteolin significantly reduced these scores. (D) Representative mIF staining of mouse psoriasis tissues. RHCG (red), KRT16 (gold), DSC2 (pink), LAMP3 (green), and DAPI (blue) in individual and merged channels are shown (*n* = 6). Scale bar, 50 μm. Luteolin treatment significantly reduced the expression of RHCG, KRT16, and LAMP3 and restored the expression of DSC2. (E) Higher‐magnification view forms (D) highlight the direct physical proximity between abnormal KCs (marked by RHCG and KRT16) and activated DCs (marked by LAMP3), which was unique to the IMQ group. Due to negligible expression of RHCG and LAMP3 in both the blank and luteolin treatment groups, only the IMQ and MTX groups are shown for comparison. Scale bar, 20 μm. (F) The percentage of DSC2‐positive (DSC2^+^) cells and LAMP3‐positive (LAMP3^+^) cells was quantified using the CellProfiler platform. The bar chart on the right shows the differences in DSC2^+^cells and LAMP3^+^cells among different groups, again demonstrating the inhibitory effect of luteolin on activated DCs and its restorative effect on DSC2 expression. Statistical analysis: Data are presented as mean ± SD, and statistical significance was assessed using one‐way ANOVA with Tukey’s post hoc test. Exact *p* values (or significance levels) are indicated on the figure.

**Figure:**
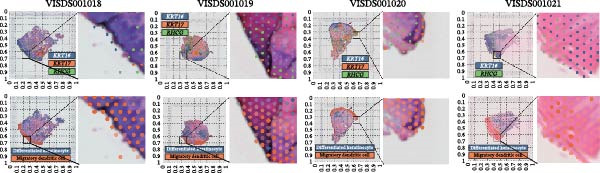
(A)

**Figure:**
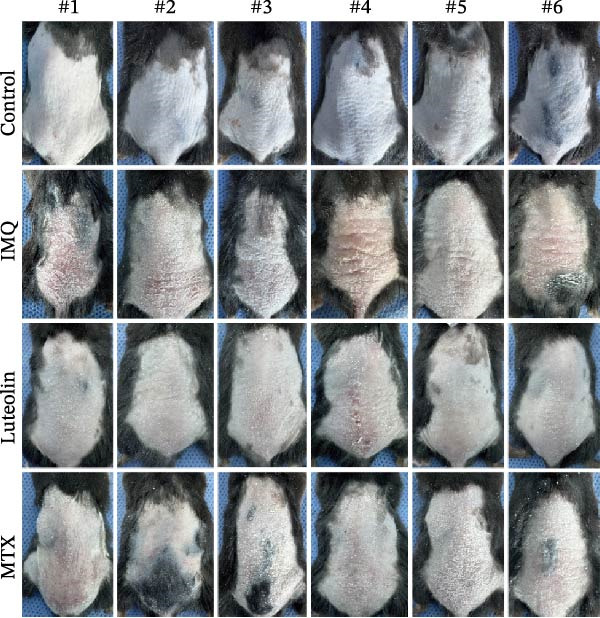
(B)

**Figure:**
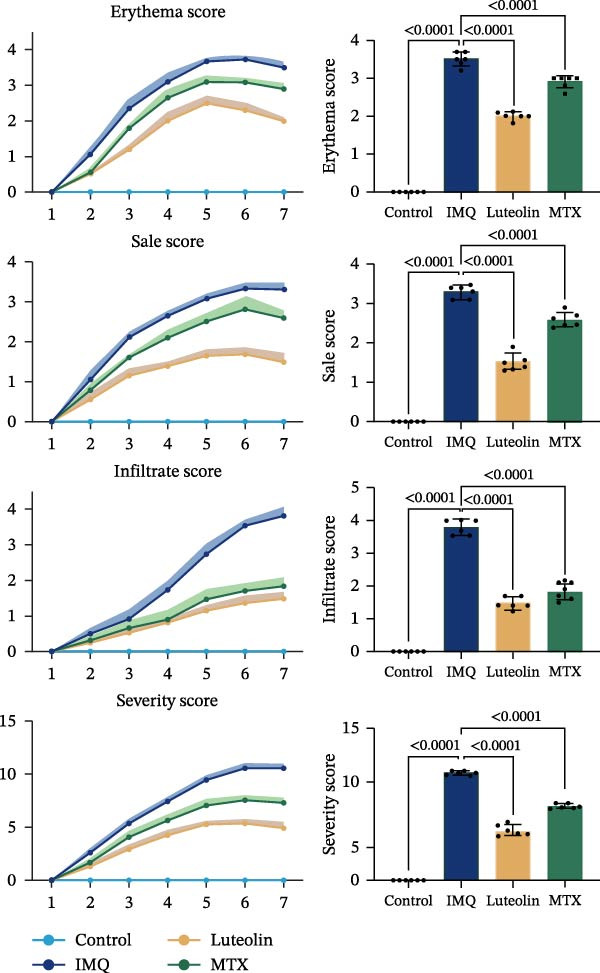
(C)

**Figure:**
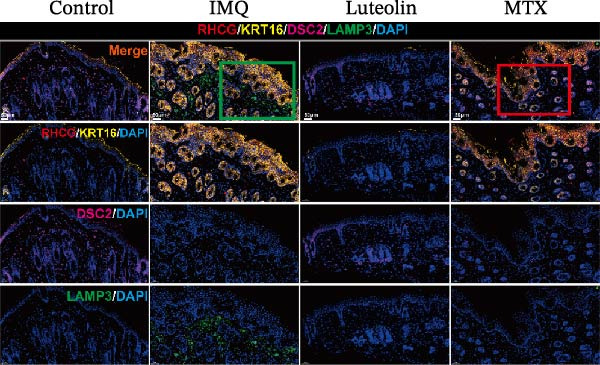
(D)

**Figure:**
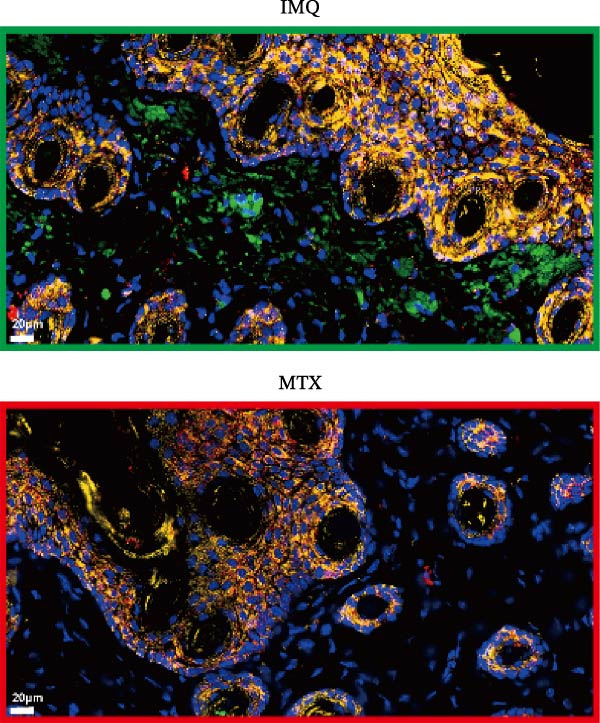
(E)

**Figure:**
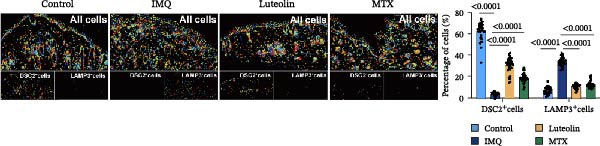
(F)

Following application of IMQ, pronounced phenotypic changes were observed in the model group, characterized by the rapid development of erythema, scaling, and significant skin thickening within 2 days, contrasting sharply with the control group. In contrast, luteolin‐treated mice showed substantial mitigation of psoriatic lesion symptoms (Figure 7B). Notably, luteolin administration also resulted in a reduced PASI score relative to the model group, achieving efficacy comparable to MTX treatment (Figure 7C).

We further evaluated the expression of RHCG, KRT16, and DSC2 via immunofluorescence staining. Consistent with our expectations, the model group displayed upregulation of KRT16 and RHCG and downregulation of DSC2 compared to controls, while both luteolin and MTX treatments counteracted these alterations (Figure 7D). Given the prevalence of DCs in luteolin‐targeting regions, we also assessed LAMP3 expression. LAMP3 levels were elevated in the model group relative to controls but were suppressed following either luteolin or MTX treatment (Figure 7D).

Of particular interest, the physical proximity between KCs and DCs—readily observable in the model group—was markedly reduced in MTX‐treated mice and nearly absent in the luteolin and control groups, likely due to diminished infiltration of both cell types (Figure 7E). To quantitatively assess activated DCs and desmosome‐expressing KCs, we employed CellProfiler to measure the proportions of LAMP3^+^cells and DSC2^+^cells across groups. As summarized in Figure 7F, both luteolin and MTX significantly decreased LAMP3^+^cell infiltration and restored DSC2 expression in KCs compared to the model group.

## 4. Discussion

TCM has long been recognized for its holistic and multitargeted approach to managing psoriasis, particularly mild‐to‐moderate cases. Formulations like CBDF exhibit efficacy by modulating inflammatory pathways such as NF‐κB and STAT3 [27, 71]. Our research identified luteolin, a flavonoid compound derived from CBDF, as a key bioactive component with potent anti‐inflammatory and anti‐proliferative properties. Previous research demonstrates that luteolin ameliorates psoriasis‐like lesions in animal models by suppressing pro‐inflammatory cytokines (e.g., IL‐17, IL‐23, and TNF‐α) and inhibiting NF‐κB activation [72, 73]. Its ability to attenuate KC hyperproliferation and immune cell infiltration positions luteolin as a promising candidate for targeted psoriasis therapy.

We previously identified RHCG as a critical pathogenic driver in psoriasis [25], with elevated expression in psoriatic KCs linked to dysregulated differentiation and cytokine secretion [25, 26]. Mechanistically, RHCG is known to drive core inflammatory responses in KC, including the activation of NF‐κB signaling pathways [28]. As these pathways are also known targets of luteolin, our findings thus propose that modulating RHCG represents a key mechanism through which luteolin exerts its anti‐inflammatory effects [74], without excluding other potential pathways. Importantly, while previous studies defined RHCG as a pathogenic driver, our work provides direct evidence that this pathway is pharmacologically targetable by a defined small molecule.

Building on this, we combined molecular docking, molecular dynamics simulations, and CETSAs to support a direct interaction between luteolin and RHCG. Consistent with this, luteolin treatment was associated with reduced RHCG abundance and dampened downstream inflammatory outputs in KCs. Together with recent reports implicating RHCG in psoriatic inflammation, these data support RHCG as a functionally relevant node for luteolin’s anti‐inflammatory effects in this context.

The interplay between KCs and DCs is central to psoriasis pathogenesis [75]. Our prior work established that RHCG amplifies KC inflammation and promotes DC activation via CXCL14–CXCR4 signaling—a pathway implicated in psoriatic plaque formation and immune cell recruitment [26]. Given that RHCG expression is regulated by hypoxia [26], variations in tissue oxygen levels could serve as an upstream signal that potentiates the KC–DC chemokine communication loop in psoriatic inflammation [76, 77]. Chemokine signaling is pivotal in orchestrating the inflammatory cascade characteristic of psoriasis [78]. Ligands such as CXCL8, CCL20, and CXCL1, interacting with their cognate receptors, direct the trafficking of key immune cells—neutrophils, Th17 cells, and DCs—into psoriatic lesions [79]. This sustained chemotaxis perpetuates a pro‐inflammatory feedback loop, driving KC hyperproliferation and the hallmark epidermal hyperplasia [80]. Consequently, targeting specific chemokine axes presents a promising therapeutic strategy for mitigating pathological inflammation in this disease [81]. Here, we demonstrate that luteolin‐mediated RHCG inhibition disrupts this axis: luteolin reduces CXCL14 secretion from KCs, impairing DC maturation in coculture systems. This aligns with broader literature emphasizing CXCL14’s role in chemoattracting immune cells and sustaining psoriatic inflammation [82]. By targeting RHCG, luteolin effectively uncouples the KC–DC communication loop, highlighting its potential to interrupt disease‐propagating signals.

Psoriatic lesions are organized into spatially distinct microdomains (niches), where KC–DC interactions drive localized inflammation [60, 83]. Our ST analysis revealed that luteolin preferentially targets DC–enriched niches, reducing their cellular density and inflammatory signature. Importantly, these niches exhibited activated desmosome signaling. Our study found that although DSC2 was transcriptionally upregulated in psoriatic lesions, its protein expression was suppressed, suggesting that posttranslational mechanisms, such as protease‐mediated degradation, may underlie desmosomal dysfunction in psoriasis, potentially exacerbated by RHCG–driven inflammatory signaling. It is well established that desmosomal proteins are significantly downregulated in psoriatic skin [84, 85], contributing to impaired epidermal cohesion and hyperproliferation, likely due to localized pro‐inflammatory cytokines such as TNF‐α and IL‐17 [86, 87], which suppress key desmosomal components, alongside elevated proteolytic activity that further disrupts junctional integrity [88]. Importantly, we observed a positive correlation between *RHCG* and *MMP9*, *KLK*5, and *KLK7*, suggesting that protease‐mediated degradation may be a key regulatory mechanism in the posttranslational control of desmosomal components in psoriasis. Notably, luteolin not only normalized DSC2 protein levels in vivo but also restored the KC–DC spatial architecture, reducing the physical proximity of these cells in murine models. This dual action, modulating both inflammation and structural components—underscores luteolin’s capacity to reverse tissue‐level pathology.

Despite these insights, our study has several limitations. First, the IMQ–induced mouse model only partially recapitulates human psoriasis pathophysiology, underscoring the need for validation in humanized models or clinical samples. Second, although we identified DSC2 dysregulation, the precise mechanistic link between RHCG and posttranslational desmosomal degradation remains unclear. Third, luteolin was administered intraperitoneally in our proof‐of‐concept study to ensure controlled exposure; however, this route limits direct clinical translation for a localized disease such as psoriasis. The pharmacokinetic profile of luteolin also requires optimization, ideally through advanced delivery strategies. Encouragingly, recent studies utilizing topical luteolin nanoformulations in similar IMQ models have reported enhanced skin retention and efficacy, supporting the development of topical or transdermal systems [89]. Future work will, therefore, prioritize optimizing local delivery approaches and conducting direct comparisons of topical versus systemic administration in terms of pharmacokinetics and efficacy. Besides, our computational method has limitations. The 10  ns MD simulation, while indicative is shorter than current standards for robust stability assessment. The binding energy of −6.0 kcal/mol suggests moderate affinity, consistent with luteolin being a natural product modulator. Although supported by CETSA, future studies using surface plasmon resonance (SPR) are warranted to quantify binding affinity (*K*_*d*_) precisely. Finally, the potential contribution of other CBDF components to luteolin’s effects should be investigated to clarify possible synergistic interactions. Subsequent studies will also focus on elucidating RHCG’s role in desmosomal turnover and evaluating luteolin’s efficacy in combination with existing biologic therapies.

## 5. Conclusion

Luteolin emerges as a multifaceted agent targeting RHCG to disrupt KC–DC cross talk, reprogram inflammatory spatial domains, and restore epidermal integrity. By bridging TCM–derived compounds with modern omics approaches, this study provides a mechanistic foundation for luteolin‐based interventions in psoriasis.

## Author Contributions

Qian Zhang and Yan‐wei Gao performed all experiments and wrote the manuscript. Cheng‐cheng Feng and Liang Yang collected clinical specimens and provided the respective clinical information. Fang Chen, Shun Guo, Yuan‐jie Liu, and Qiu‐ya Lu provided relevant experimental facilities and technical support. Chen Ji and Hui Shen developed the experimental plan and provided the research funding. All authors contributed to data analysis and manuscript preparation.

## Funding

This study was supported by the National Natural Science Foundation for Young Scientists of China (Grant 82205113), the Jiangsu Province Traditional Chinese Medicine Science and Technology Development Plan General Project (Grant MS2023102), and the Suzhou Applied Basic Research (Medical and Health) Technology Innovation Project (Grant SYWD2024047).

## Disclosure

All authors have reviewed the manuscript and approved the final manuscript for submission.

## Ethics Statement

The study protocol was reviewed and approved by the Ethics Committee of Zhangjiagang TCM Hospital Affiliated to Nanjing University of Chinese Medicine (Approval Number 2024NL‐077‐01; approved on 2024‐03‐28). Written informed consent was obtained from participating clinicians and patients. All procedures complied with the Declaration of Helsinki.

## Consent

Written consent for publication of personal data was obtained from all patients and/or their legal guardians.

## Conflicts of Interest

The authors declare no conflicts of interest.

## Supporting Information

Additional supporting information can be found online in the Supporting Information section.

## Supporting information


**Supporting Information 1** Table S1 lists the reagents, drugs, and antibodies used in this study. Figure S1 validates the transfection efficiency.

**Supporting Information 2** Tables S2–S9 provide luteolin candidate targets predicted by six platforms and the deduplicated set (564 unique targets). Table S10 summarizes the CABS‐flex ensemble models and per‐residue RMSF values derived from the docked PDB structure.

**Supporting Information 3** The “Uncut blot” file contains the original, uncropped western blot images for Figures 2K, 2L, 3H, and 3K, with sample labels and molecular‐weight markers.

## Data Availability

We confirm that all the data reported in this article are authentic and valid and can be shared upon reasonable request. Data and related materials are available from the corresponding author, Hui Shen (zjgzy046@njucm.edu.cn).
