# Supplementary material for: Luteolin Disrupts Keratinocyte–Dendritic Cell Communication in Psoriasis by Targeting Rh Family C Glycoprotein
Source: Mediators Inflamm. 2026 Mar 12;2026:9564209. doi: 10.1155/mi/9564209 (PMC13140231; doi:10.1155/mi/9564209)
Supplement: Supplementary file 1 — Supporting Information 1 Table S1 lists the reagents, drugs, and antibodies used in this study. Figure S1 validates the transfection efficiency. [file MI-2026-9564209-s001.docx]

## Table S1 Reagents, Drugs, and Antibodies Used in This Study

| Antibodies and Reagents | Manufacturer, Country, Cat number, Lot number | Concentration |
| --- | --- | --- |
| Human Peripheral Blood Maturation Dendritic cell Complete Medium | Procel, China, CM-H179B, WH4025E221 | - |
| Dulbecco’s modified Eagle’s medium (DMEM) | cytiva, USA, SH30022.0., AL30835543 | - |
| Phosphate buffered saline (PBS) | cytiva, USA, SH30256.01, AK30794646 | - |
| Fetal bovine serum（FBS） | Gibco, USA, 10099141C, A2689435CP |  |
| Trypsin | KeyGEN, China, KG2101-100, 20250528 | - |
| Penicillin-Streptomycin Solution | Procell, China, PB180120, WHAB25C031 | - |
| Cell Counting Kit-8 | DOJINDO, Japan, CK04-3000tests, WA791 | - |
| Corning® Transwell®-Clear Inserts, Polyester (PET) membrane | Corning, USA, 3460, 22324006 | - |
| GL-RHCG | GeneChem, China, KL65969-1GL, 10109193 | - |
| PL-RHCG | GeneChem, China, KL65969-1PL, 10109193 | - |
| Lipofectamine 3000 | Invitrogen, USA, L3000015, 3034178 | - |
| Opti-MEM | Gibco, USA, 31985-070, 3096260 | - |
| Bicinchoninic acid (BCA)  Assay Kit | ThermoFisher, USA, A55860, ZG39391A | - |
| Anti-β-actin Rabbit polyclonal Antibody | Cell Signaling Technology, USA,4970s, 19 | WB 1:1000 |
| GAPDH ab（HRP） | Abways, China, AB2000, F300801 | WB 1:1000 |
| Anti-RHCG polyclonal Antibody | Abcam, USA, AB106837, 1116800-1 | WB 1:1000 |
| Anti-Cytokeratin1/K1 Rabbit polyclonal Antibody | Abcam, USA, AB185628, 1023500-27 | WB 1:1000 |
| Anti-Cytokeratin16/K16 Rabbit polyclonal Antibody | Abcam, USA, AB7646, 1053991-6 | WB 1:1000 |
| Anti-s100a12/cgrp Rabbit polyclonal Antibody | Abcam, USA, AB272713, 1010805-21 | WB 1:1000 |
| Anti-Hexokinase II Rabbit polyclonal Antibody | Abcam, USA, ab104836, 1035842-5 | WB 1:1000 |
| LAMP3 Polyclonal antibody | Proteintech, USA, 12632-1-AP, 00155327 | WB 1:1000 |
| Secondary Antibody Dilution Buffer | Beyotime, China, P0023D-500 | - |
| QuickBlock™ Primary Antibody Dilution Buffer for Western Blot) | Beyotime, China, P0256-500 | - |
| QuickBlock™ Blocking Buffer for Western Blot | Beyotime, China, P0252-500 | - |
| Methotrexate | TargetMol, China, T1485, 146273 | - |
| Luteolin | MCE, China, HY-N0162, 129620 | - |
| Human IL-17A Recombinant Protein, PeproTech® | Gibco, USA, 200-17-25UG, 112384C0624 | - |
| Human IL-22 Recombinant Protein, PeproTech® | Gibco, USA, 200-22-25UG, 0710246I1924 | - |
| Human TNF-alpha Recombinant Protein, PeproTech® | Gibco, USA, 300-01A-50UG, 031825F1724 | - |
| Recombinant Human IL-1 alpha Protein | ABclonal, China, RP00098, 9633110303 | - |
| Recombinant Human Oncostatin-M/OSM Protein | ABclonal, China, RP00054, 9633021701 | - |
| Protease and phosphatase inhibitor cocktail for general use, 50X | Beyotime, China, P1045, 20261231 | - |
| RIPA Lysis Buffer | Beyotime, China, P0013C, A167250311 | - |
| Omni-Easy™Protein Sample Loading Buffer(Denaturing,Reducing,5×） | EpiZyme, China, LT101, 039627000 | - |
| ColorMixed Protetin Marker 180（10-180kDa） | Abclonal, China, RM19001, 9625325B21 | - |
| Tricolor Prestained Protein Marker（10-250 kDa） | EpiZyme, China, EpiZyme, WJ103, 027352000 |  |
| QuickBlock™ Blocking Buffer for Western Blot | Beyotime, China, P0252-500, A196250327 | - |
| Tris-MOPS-SDS Running Buffer Powder | Genscript, China, M00138, C31382407 |  |
| SurePAGE, Bis-Tris | Genscript, China, M00669, C3535240 |  |
| 1× Ice-free bath fast film transfer buffer | Servicebio, China, G2148-1L, GC2501010 |  |
| NCM Western Blot Stripping Buffer | NCM Biotech, China, WB6200, 20250318 |  |
| Highly Cross-Adsorbed Goat (Polyclonal) Anti-Mouse IgG(H+L) Antibody | LI-COR, USA, 926-68070, Q04695 | WB 1:5000 |
| Highly Cross-Adsorbed Goat (Polyclonal) Anti-Rabbit IgG(H+L) Antibody | LI-COR, USA, 926-68071, S11385 | WB 1:5000 |
| Human BRAK/CXCL14 ELISA KIT | Fenxi Biotechnology Co., Ltd, China, FXs05941 |  |
| Lactic acid content detection kit | Fenxi Biotechnology Co., Ltd, China, FXs-z0001 |  |
| ImiquimodCream | iNova Pharmaceuticals (Singapore) Pte. Limited, Australia, H20160079, 7549 |  |

**Figure S1**

**
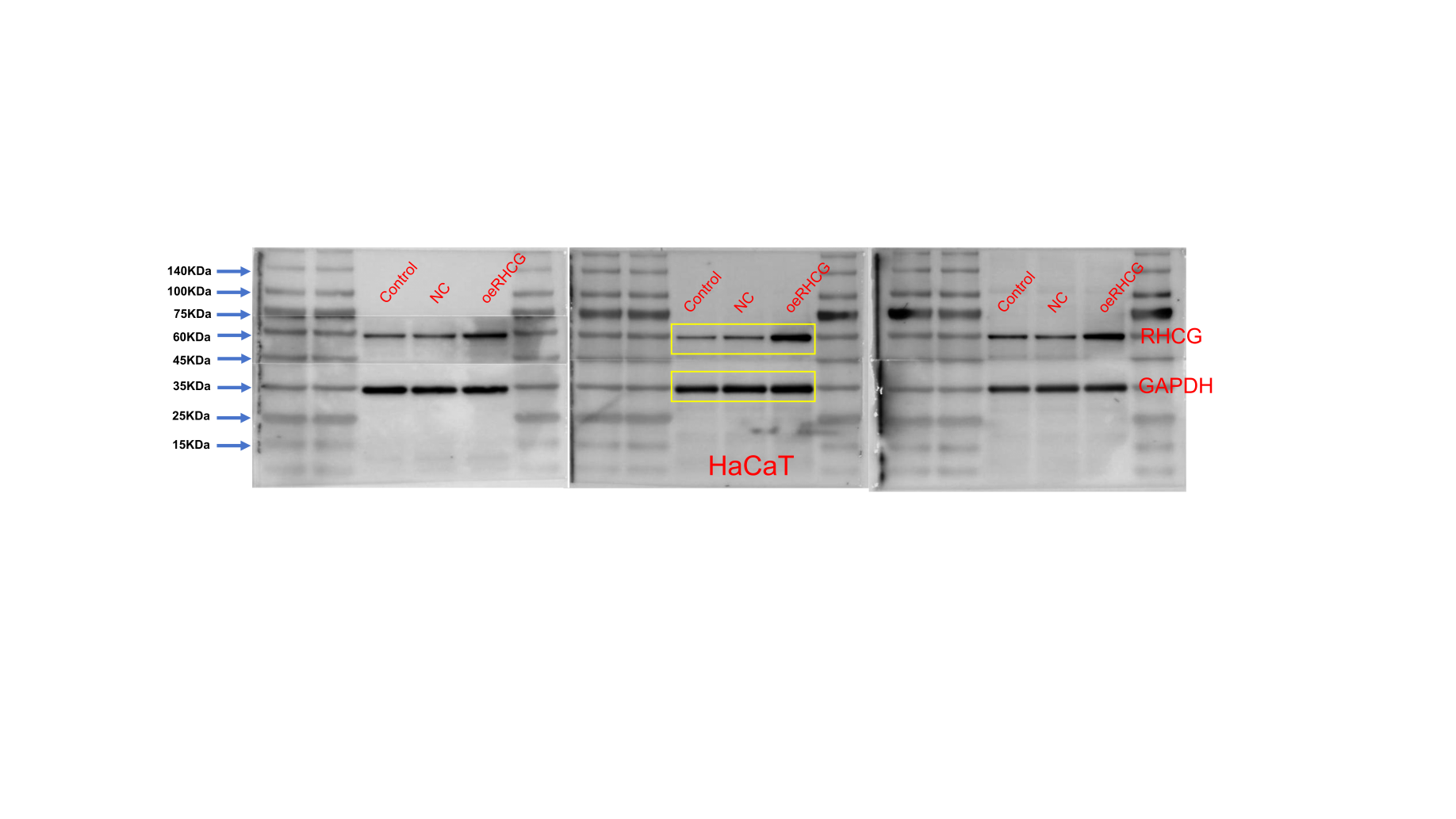
**

**Figure S1 Validation of transfection efficiency**
