# Supplementary material for: Luteolin Disrupts Keratinocyte–Dendritic Cell Communication in Psoriasis by Targeting Rh Family C Glycoprotein
Source: Mediators Inflamm. 2026 Mar 12;2026:9564209. doi: 10.1155/mi/9564209 (PMC13140231; doi:10.1155/mi/9564209)

Figure 2K uncut blot

ladder maker: ColorMixed Protetin Marker 180（10-180kDa）, Abclonal，cat：RM19001，lot:9625325B21

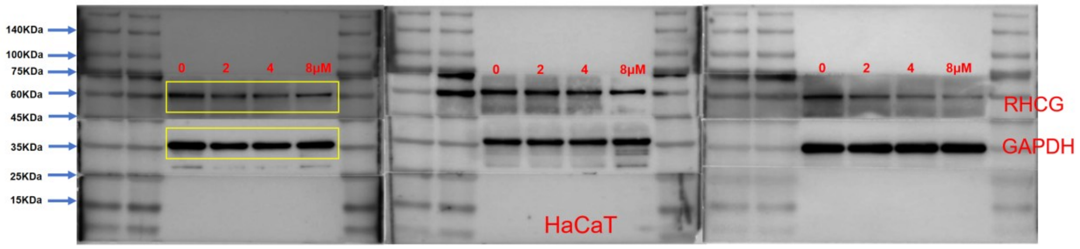

Figure 2L uncut blot

ladder maker: Tricolor Prestained Protein Marker（10-250 kDa） EpiZyme, cat：WJ103, lot: 027352000

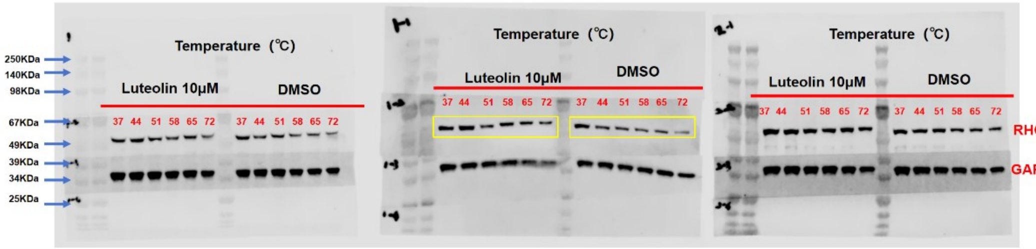

Figure 3H uncut blot

ladder maker: ColorMixed Protetin Marker 180（10-180kDa）, Abclonal，cat：RM19001，lot:9625325B21

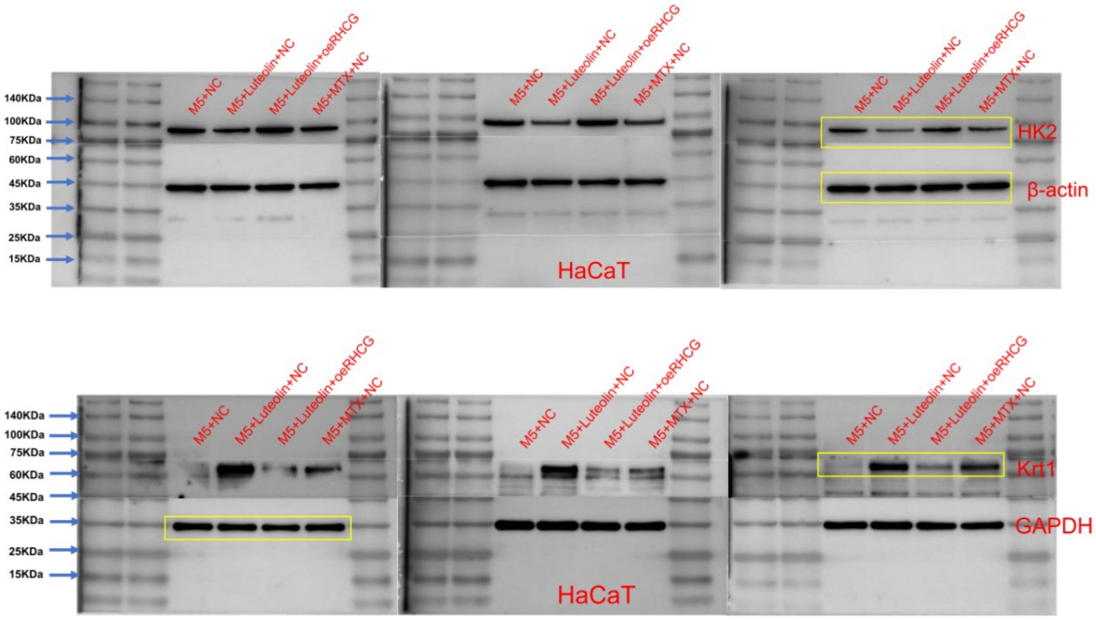

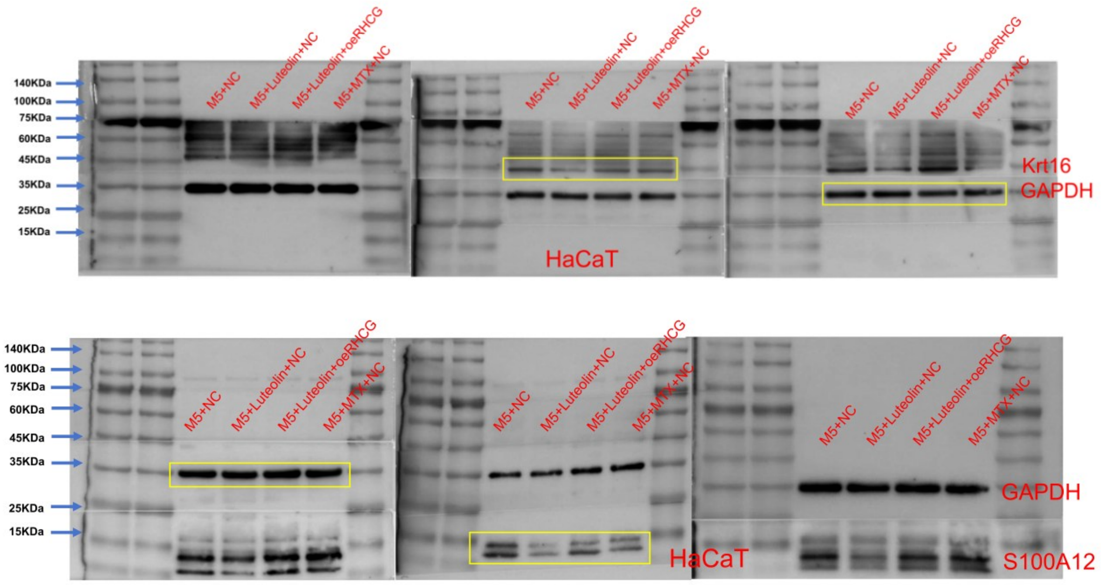

Figure 3K uncut blot

ladder maker: ColorMixed Protetin Marker 180（10-180kDa）, Abclonal，cat: RM19001，lot:9625325B21

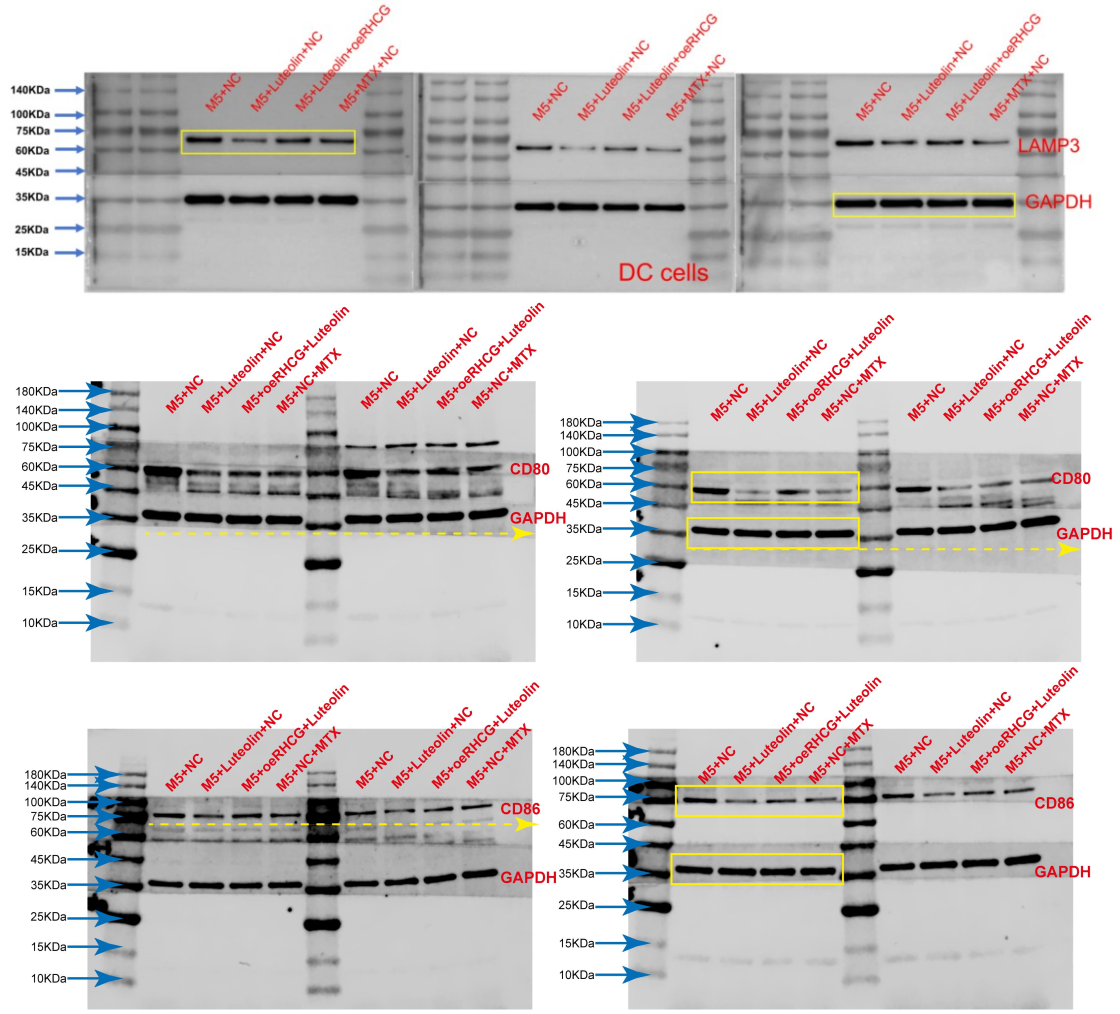

Supplement: Supplementary file 3 — Supporting Information 3 The “Uncut blot” file contains the original, uncropped western blot images for Figures 2K, 2L, 3H, and 3K, with sample labels and molecular‐weight markers. [file MI-2026-9564209-s002.pdf]
